# The Chaperone BAG6 Regulates Cellular Homeostasis between Autophagy and Apoptosis by Holding LC3B

**DOI:** 10.1016/j.isci.2020.101708

**Published:** 2020-10-21

**Authors:** Yuanyuan Chu, Xingqi Dong, Yingjin Kang, Jingnan Liu, Tao Zhang, Cuiwei Yang, Zhangshun Wang, Wangchen Shen, Huanhuan Huo, Min Zhuang, Junxia Lu, Yanfen Liu

**Affiliations:** 1 School of Life Science and Technology, ShanghaiTech University, Shanghai 201210, China; 2 CAS Center for Excellence in Molecular Cell Science, Shanghai Institute of Biochemistry and Cell Biology, Chinese Academy of Sciences, Shanghai 200031, China; 3 University of Chinese Academy of Sciences, Beijing 100049, China

**Keywords:** Biological Sciences, Biochemistry, Molecular Biology, Cell Biology

## Abstract

AMFR/gp78 and USP13 are a pair of ubiquitin ligase and deubiquitinase that ensure the accuracy of endoplasmic reticulum-associated degradation (ERAD). Depletion of USP13 leads to caspase activation and cleavage of the ERAD chaperone BAG6, which is reversed by knockdown of *AMFR*. However, the mechanism and physiological relevance of this regulation are still unclear. Here, by using the NEDDylator system, we screened out TXN as a substrate of AMFR and USP13 and showed its involvement in regulating CASP3 activation and BAG6 cleavage. Furthermore, we showed that the cleaved N-terminal BAG6 is located in the cytosol and interacts with both LC3B-I and unprocessed form of LC3B (Pro-LC3B) through the LIR1 motif to suppress autophagy. An NMR approach verified the direct interaction between BAG6 LIR1 and LC3B-I or Pro-LC3B. Collectively, our findings uncover a mechanism that converts BAG6 from an ERAD regulator to an autophagy tuner and apoptosis inducer during ER stress.

## Highlights

• AMFR and USP13 regulate CASP3-mediated BAG6 cleavage
• CASP3 cleaves BAG6 and the cleaved N-terminal BAG6 binds to LC3B-I
• BAG6 binds to LC3B-I through its LIR1 motif to inhibit autophagosome formation
• BAG6 regulates cellular homeostasis between autophagy and apoptosis

## Graphical Abstract

**Figure:**
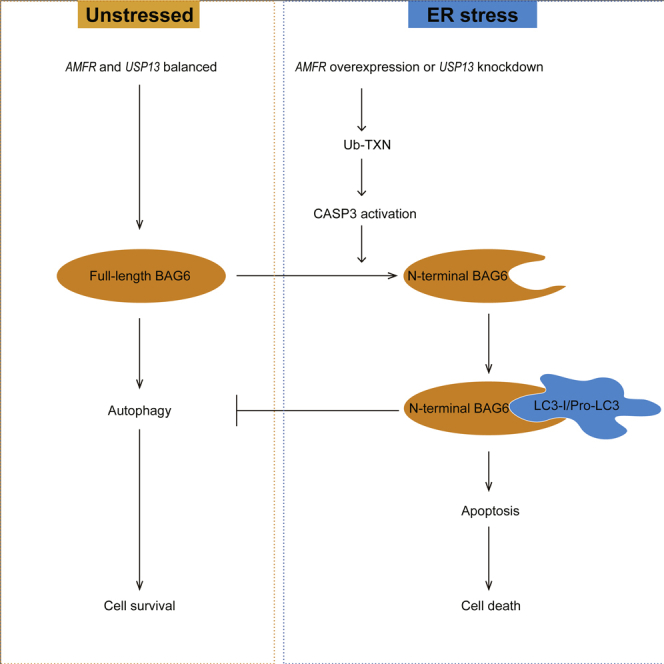

## Introduction

In eukaryotic cells, endoplasmic reticulum-associated degradation (ERAD) plays a crucial role in maintaining cellular homeostasis by monitoring and degrading misfolded or unfolded proteins in the ER (Brodsky, 2012; Preston and Brodsky, 2017; Ruggiano et al., 2014; Smith et al., 2011). Dysfunction in the ERAD pathway can lead to irreversible ER stress, which eventually activates cellular apoptosis pathway (Hetz, 2012; Hetz and Papa, 2018; Tabas and Ron, 2011). Various E3 ubiquitin ligases selectively recognize these different aberrant substrates. At the same time, some deubiquitinases (DUBs) are also recruited to keep the specificity of modification. Among them, AMFR/gp78 and USP13 are a pair of ubiquitin ligase and deubiquitinase functioning in ERAD (Chen et al., 2006; Fang et al., 2001; Liu et al., 2014). Previously we have reported that AMFR ubiquitinates not only ERAD substrates but also ERAD machinery protein UBL4A, one of the components of BAG6 complex, leading to the disassembly of BAG6 complex. On the other hand, USP13 antagonizes AMFR by deubiquitinating UBL4A to maintain the integrity of BAG6 complex and therefore promotes ERAD (Liu et al., 2014). Previously, we and other groups have demonstrated that BAG6 is a multifunctional chaperone protein that can hold hydrophobic domain of tail-anchored (TA) membrane proteins (Leznicki et al., 2010; Mariappan et al., 2010) or misfolded proteins (Ernst et al., 2011; Hessa et al., 2011; Mariappan et al., 2010; Minami et al., 2010; Wang et al., 2011) and direct them to distinct destinations. Therefore, the integrity of BAG6 complex is necessary for holding client proteins from aggregation. Additionally, we have also found that overexpression of *AMFR* or knockdown of *USP13* results in the cleavage of BAG6 by CASP3 (Liu et al., 2014). A CASP3 cleavage site, DEQD, has been reported in the C terminus of BAG6 (Wu et al., 2004). However, the detailed mechanisms of how CASP3 is activated to cleave BAG6 in response to *USP13* depletion are still unknown.

Autophagy is a highly conserved degradation process that recycles cellular components and defends against intracellular pathogens (Bento et al., 2016; Mizushima et al., 2011). It is initiated from the biogenesis of a crescent-shaped isolation membrane (IM), named phagophore, which expands to form a double membrane vesicle called autophagosome. The autophagosome contains cellular components including damaged organelles, protein aggregates, and invasive microbes, which are degraded by the hydrolases after autophagosome fusion with lysosome (Galluzzi et al., 2014, 2017; He and Klionsky, 2009). The initiation and maturation of autophagosome are strictly regulated by autophagy-related proteins. Upon autophagy induction, the activated ULK1/ATG13/RB1CC1 kinase complex transfers to the autophagosome formation site enriched with phosphatidylinositol (PtdIns) (Karanasios et al., 2013; Nishimura et al., 2017). The ULK1/ATG13/RB1CC1 complex then activates downstream substrates including class III phosphatidylinositol 3-kinase (PtdIns3K) complex. The latter then generates PtdIns3P, which provides the platform for WIPIs docking (Dooley et al., 2014; Itakura and Mizushima, 2010; Karanasios et al., 2013). WIPI2b directly binds to ATG16L1 and recruits ATG12-5-16L1 complex to the phagophore assembly site for LC3 lipidation and membrane elongation (Dooley et al., 2014). LC3 is considered as a hallmark of autophagosome, and its covalent attachment to lipid membrane is essential for autophagosome formation and maturation (Klionsky et al., 2008). LC3 conjugation on the phagophore is essential for the phagophore expansion and fusion to form autophagosome. Interestingly, many proteins contain an LC3-interacting region (LIR), which mediates binding to LC3, and they are usually involved in regulating the autophagy signaling pathway (Alemu et al., 2012; Johansen et al., 2017; Kraft et al., 2012; Pankiv et al., 2007).

BAG6 participates in a variety of cellular processes, including protein quality control, gene regulation, and apoptosis (Lee and Ye, 2013). BAG6 was initially found as an apoptosis regulator by interacting with Reaper, a central apoptosis effector in *Drosophila* (Thress et al., 1998, Thress et al., 1999). Subsequent studies discovered BAG6 functions as an anti-apoptotic regulator and interacts with multiple apoptotic modulators (Desmots et al., 2008; Kumar et al., 2004; Minami et al., 2007). Depletion of *Bag6* in mouse causes cell death and proliferation defects, resulting in embryonic lethality (Desmots et al., 2005). Besides, BAG6 is necessary for DNA damage-induced apoptosis by controlling the TP53/p53 signaling pathway in human cells (Sasaki et al., 2007). Interestingly, it has been recently reported that BAG6 also promotes autophagy by transporting EP300/p300 into the nucleus to acetylate TP53, and truncated BAG6 holds EP300 in the cytosol to hyperacetylate ATG7 to suppress autophagy (Sebti et al., 2014a, 2014b). Therefore, it seems that BAG6 may function in both apoptotic and autophagic pathways. We then questioned whether BAG6 is a switcher of autophagy and apoptosis during ER stress.

Here, by using a NEDDylator system, we identified the substrates of AMFR and USP13, which might mediate the activation of CASP3 and consequence of BAG6 cleavage. Based on mass spectrometry analysis, we have identified multiple substrates and demonstrated TXN (thioredoxin) as a substrate of AMFR and USP13 involved in CASP3 activation and BAG6 cleavage. Ubiquitinated TXN accompanies CASP3 activation to cleave BAG6. Cleaved BAG6 remains in the cytosol and interacts with excessive LC3B-I or the unprocessed form of LC3B (Pro-LC3B) through the LIR1 motif to suppress autophagy. Our NMR data further verified that BAG6 binds to LC3B-I or Pro-LC3B through some specific amino acids and BAG6 binding to LC3B-I can stabilize LC3B-I structure. Therefore, our study provides evidence that ERAD machinery proteins can regulate the balance between autophagy and apoptosis.

## Results

### Depletion of *USP13* Induces Apoptosis and Suppresses Autophagy

**Figure 1:**
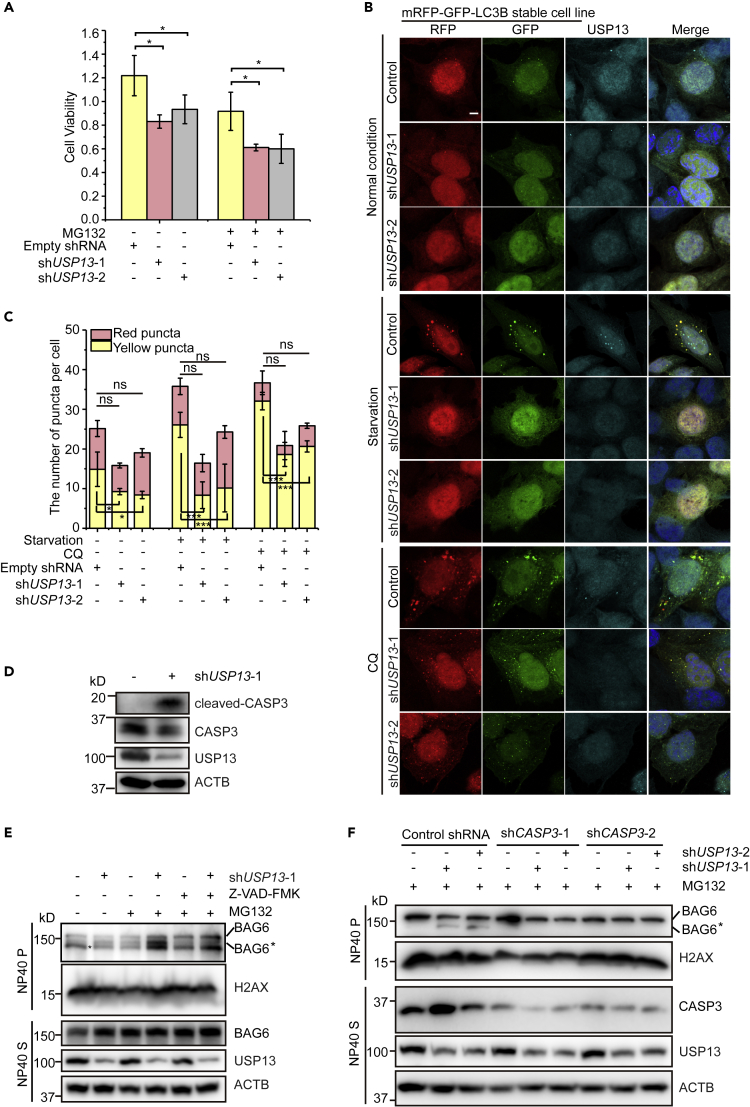
Depletion of *USP13* Induces BAG6 Cleavage and Suppresses Autophagy (A) *USP13* knockdown decreases cell viability with or without MG132 treatment. Cells were transfected with control shRNA, *USP13* shRNA-1, or *USP13* shRNA-2. Cells were treated with DMSO or MG132 (10 μM) for 12 h. Cell viability was measured by MTS assay. Data are represented as mean ± SD from three independent experiments. ∗p < 0.05 (one-way ANOVA). (B) The number of LC3B-positive puncta is less in *USP13* knockdown cells. HeLa cells stably expressing *mRFP-GFP-LC3B* were transiently transfected with control shRNA, *USP13* shRNA-1, or *USP13* shRNA-2. Cells were starved in Earle's Balanced Salt Solution (EBSS) or treated with 20 μM CQ for 4 h, and analyzed for LC3B puncta. USP13 is in cyan (Alexa 633) and nuclei are in blue. Scale bar, 5 μm. (C) Quantification of yellow (RFP^+^GFP^+^) and red (RFP^+^GFP^−^) puncta per cell as represented in Figure 1B. Data are represented as mean ± SD from three independent experiments, and more than 80 cells were scored in each experiment. ns, not significant; ∗p < 0.05; ∗∗∗p < 0.001 (one-way ANOVA). (D) *USP13* knockdown induces CASP3 cleavage and activation. Cells were transfected with control shRNA or *USP13* shRNA-1, and the samples were immunoblotted with anti-CASP3 and anti-cleaved-CASP3 antibodies. (E) The membrane fraction BAG6 is more cleaved in response to *USP13* depletion induced by MG132 and blocked by Z-VAD-FMK. Cells transfected with control shRNA or *USP13* shRNA-1 were treated with DMSO, MG132 (10 μM), or MG132 (10 μM) plus Z-VAD-FMK (40 μM) for 12 h. Cells were then subjected to sequential extraction by NP40- and SDS-containing buffers. In the membrane fraction, more cleaved BAG6 was detected in *USP13* knockdown cells than that in the control cells. BAG6∗ represents cleaved N-terminal BAG6. ACTB and H2AX serve as the markers for the NP40 soluble (S) and insoluble (P) fractions, respectively. (F) Knockdown of *CASP3* inhibits membrane fraction BAG6 cleavage, which is induced by *USP13* knockdown under MG132 treatment. Cells were co-transfected with control shRNA, *USP13* shRNA-1, or *USP13* shRNA-2, with or without *CASP3* shRNA-1 or *CASP3* shRNA-2. Samples were treated with MG132 (10 μM, 12 h) before being lysed for analysis. See also Figure S1.

Impaired ERAD pathway causes prolonged ER stress, which activates alternative signal pathways and promotes apoptosis (Hetz, 2012; Hetz and Papa, 2018; Tabas and Ron, 2011). *USP13* knockdown results in loss of ER homeostasis and prolonged ER stress, so we examined whether *USP13* depletion activates apoptosis in cells. As shown in Figure 1A, the cell viability was decreased in *USP13* knockdown cells, and the knockdown cells were tended to apoptosis compared with the control cells. MG132 is a proteasome inhibitor and has been shown to induce apoptosis (Emanuele et al., 2002; Giuliano et al., 1999; Lauricella et al., 2003; MacLaren et al., 2001; Rock et al., 1994). It is easier to detect the apoptotic change in cells with MG132 treatment. In our experiment, with MG132 treatment, the cell viability of both the control and *USP13* knockdown cells was significantly decreased (Figure 1A). Because apoptosis and autophagy are two pathways intertwined in cells to control cell fate, we examined whether USP13 could regulate autophagy. To monitor the autophagic flux, we employed a HeLa cell line stably expressing *mRFP-GFP-LC3B* (Yan et al., 2018). The autophagosomes bearing LC3B appeared as yellow puncta (with both GFP and RFP signals), but once fused with lysosomes, the autolysosomes were detected as red puncta (with only RFP signal in lysosome) because green fluorescence signal was quenched by low pH. Interestingly, we found that, under both normal and starvation (EBSS treatment) conditions, *USP13* knockdown substantially reduced the number of yellow puncta (autophagosomes) (Figures 1B and 1C). In contrast, there was no significant difference in the number of red puncta (autolysosomes) between the control and *USP13* knockdown cells (Figure 1C). These data suggest that *USP13* knockdown may suppress the early step of autophagy. Chloroquine (CQ) is a drug that inhibits the fusion of autophagosome with lysosome, which leads to the accumulation of autophagosomes in cells. It is commonly used to measure changes in autophagic flux. The accumulation of yellow puncta was reduced in *USP13* knockdown than in the control cells under CQ treatment (Figures 1B and 1C), suggesting that *USP13* knockdown blocks autophagic flux at an early stage. Therefore, depletion of *USP13* promotes cellular apoptosis and impedes autophagy.

We showed previously that knockdown of *USP13* promotes the cleavage of BAG6. This phenotype is further enhanced by treating cells with the proteasome inhibitor MG132 (Liu et al., 2014). To explore the physiological function of this regulation, we first identified the protease responsible for BAG6 cleavage under these conditions. Since the cleavage site is predicted to be close to the C terminus, which harbors a known caspase cleavage site, we tested whether BAG6 cleavage induced by *USP13* depletion could be due to the activation of caspase. Interestingly, we found that CASP3 was robustly cleaved and activated in *USP13* knockdown cells compared with that in control cells (Figure 1D). We then tested BAG6 cleavage in the control and *USP13* knockdown cells. Cells were solubilized by an NP40-containing lysis buffer. We analyzed both the NP40-soluble and -insoluble fractions by immunoblotting, because we found previously that cleaved BAG6 preferentially accumulates in NP40-insoluble fractions. As expected, knockdown of *USP13* generated a fast migrating BAG6 species, which was more accumulated in the presence of MG132 but was blocked with the treatment of a pan-caspase inhibitor Z-VAD-FMK (Figure 1E). It was reported previously that MG132 treatment results in CASP3 and CASP7 activation (Emanuele et al., 2002; Giuliano et al., 1999; Gray et al., 2010; Lauricella et al., 2003). The treatment of MG132 in our assay is to magnify the effect of caspase activation. It is worth pointing out that, with MG132 treatment, in some cases BAG6 is also cleaved even in the control cells; however, the amount is substantially less than that in *USP13* knockdown cells. Similar observation was obtained in CRISPR cells that have *USP13* completely knocked out (Figure S1A). The above results further support the conclusion that *USP13* knockdown-induced BAG6 cleavage is dependent on caspase activity. Because CASP2, CASP3, and CASP7 are known caspases recognizing the predicted cleavage site in BAG6, we knocked down each of these caspases in *USP13*-depleted cells. As expected, individual knockdown of these caspases rescued BAG6 cleavage in *USP13* knockdown cells (Figures 1F, S1B, and S1C). *In vitro* assay using purified proteins further confirmed the cleavage activity of all three caspases toward BAG6, with CASP3 and CASP7 having a higher activity compared with CASP2 (Figure S1D). A single mutation introduced at the cleavage site (D995A) completely abolished cleavage by the caspases (Figure S1E). These results suggest that *USP13* depletion activates caspases and induces BAG6 cleavage.

### A NEDDylator System to Screen AMFR Substrates

**Figure 2:**
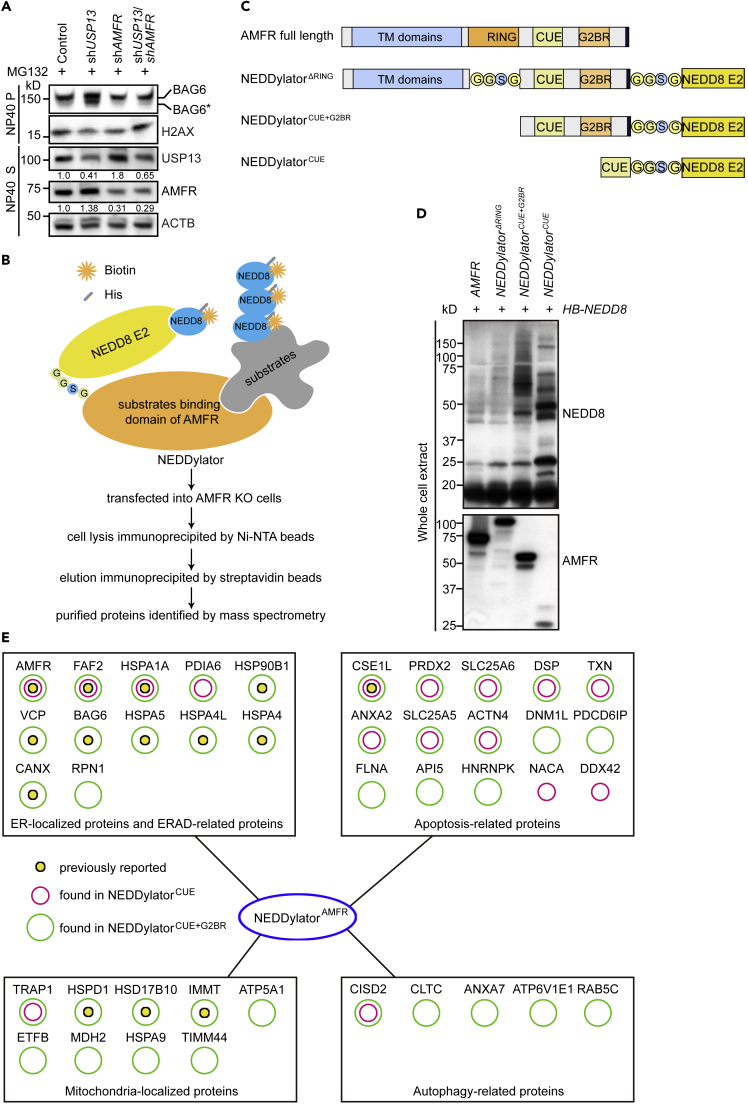
Screening of AMFR Substrates by the NEDDylator System (A) The membrane fraction BAG6 is more cleaved in response to *USP13* knockdown and is rescued by co-transfecting with *AMFR* shRNA. Cells were transfected with control shRNA, *USP13* shRNA-1, *AMFR* shRNA-1, or (*USP13* shRNA-1 + *AMFR* shRNA-1) and were treated with MG132 (10 μM, 12 h). Samples were prepared using the same procedure as in Figure 1E and were immunoblotted with the indicated antibodies. (B) Schematic overview of the NEDDylator screening system. The E3 *AMFR* fragment was fused with NEDD8 E2 *UBE2M* to generate the NEDDylator. The NEDDylator together with the His-biotin fused *NEDD8* (*HB-NEDD8*) were co-transfected into *AMFR* knockout cells. Cells were treated with biotin (5 μM) for 48 h. After that, cell lysis was immunoprecipitated by Ni-NTA beads. The elusion contained proteins covalently conjugated with HB-NEDD8 and was further immunoprecipitated by streptavidin beads. The final purified proteins were identified by mass spectrometry. (C) Domain structure of AMFR and the three NEDDylators generated based on different AMFR fragments. (D) Comparison of the NEDDylation efficiency of the three *NEDDylators*. *AMFR* knockout cells were transfected with *HB-NEDD8*, together with *AMFR* or the *NEDDylators*. Whole-cell extract was immunoblotted with an anti-AMFR antibody to indicate the successful expression of NEDDylators, and an anti-NEDD8 antibody to show NEDDylation efficiency of different NEDDylators. (E) Mass spectrometry analysis of the substrates of NEDDylator^CUE^ and NEDDylator^CUE+G2BR^. High-confidence candidate substrates of AMFR that could potentially activate caspases were selected from the whole profile and categorized into four groups based on the signal pathways they involved. See also Figure S2 and Table S1.

We previously reported that USP13 antagonizes E3 AMFR-mediated ubiquitination in the ERAD pathway. Interestingly, overexpression of *AMFR* also promotes BAG6 cleavage (Liu et al., 2014), whereas knockdown of *AMFR* in *USP13* knockdown cells suppresses *USP13* deficiency-induced BAG6 cleavage (Figure 2A). Thus, it seemed that the two enzymes may antagonize each other to modify substrate ubiquitination during apoptosis. To screen for such substrates, we employed a well-developed NEDDylator system, which captures the substrates of a specific E3 by conjugating the target proteins with ubiquitin-like NEDD8 molecule (Figure 2B) (Zhuang et al., 2013).

AMFR contains multiple transmembrane (TM) domains at the N terminus, followed by a RING, a CUE, a G2BR, and a VIM motif at the C terminus. The RING and G2BR domains are responsible for interacting with the ubiquitin-conjugating enzyme (E2) UBE2G2, the CUE domain recognizes ubiquitin chain on substrates, and the VIM motif interacts with VCP/p97. We first verified that AMFR itself has no NEDDylation activity (Figure S2A). To transfer NEDD8 to AMFR substrates, we designed three NEDDylators by fusing the NEDD8 E2 conjugating enzyme UBE2M with the different length of AMFR (Figure 2C). The resulting chimeras were named as NEDDylator^ΔRING^, NEDDylator^CUE+G2BR^, and NEDDylator^CUE^ (Figure 2C). We then transfected these three NEDDylators into *AMFR* knockout cells, together with His-biotin-tagged NEDD8 (HB-NEDD8). Immunoblotting analysis of the whole-cell extract showed that compared with AMFR and NEDDylator^ΔRING^, NEDDylator^CUE+G2BR^ and NEDDylator^CUE^ could efficiently promote substrate NEDDylation (Figure 2D), implying that shorter chimera yields higher efficiency. The conclusion was further verified by testing a known AMFR substrate FAF2/UBXD8. As expected, only NEDDylator^CUE+G2BR^ and NEDDylator^CUE^, but not NEDDylator^ΔRING^, could transfer NEDD8 to FAF2 (Figure S2B). Therefore, NEDDylator^CUE^ and NEDDylator^CUE+G2BR^ were used for the later experiments.

Affinity purification and mass spectrometry identified 371 proteins that were modified by NEDD8 in the presence of NEDDylator^CUE+G2BR^ and 110 proteins in the presence of NEDDylator^CUE^ (Table S1, related to Figure 2). We then selected substrates that were possible targets that activate caspases and divided them into four groups: ERAD-related proteins, mitochondria-localized proteins, apoptosis-related proteins, and autophagy-related proteins. In each group, we found substrates previously identified as AMFR-interacting proteins including AMFR itself, FAF2 and BAG6 in the ERAD pathway; HSPD1 and IMMT on mitochondrion; and CSE1L as an apoptosis-related protein, which validates our approach (Figure 2E).

### AMFR Cooperates with USP13 to Regulate the Ubiquitination of TXN

Among the identified proteins, we focused on TXN, a redox protein that can denitrosylate target proteins to regulate protein activity (Benhar et al., 2008; Mitchell et al., 2007; Rössig et al., 1999). TXN reduces S-nitrosylated CASP3 to CASP3 to activate apoptosis (Benhar et al., 2008). TXN maintains the activity of cytosolic CASP3, whereas TXN2 regulates the activity of mitochondrial CASP3 (Benhar et al., 2008).

**Figure 3:**
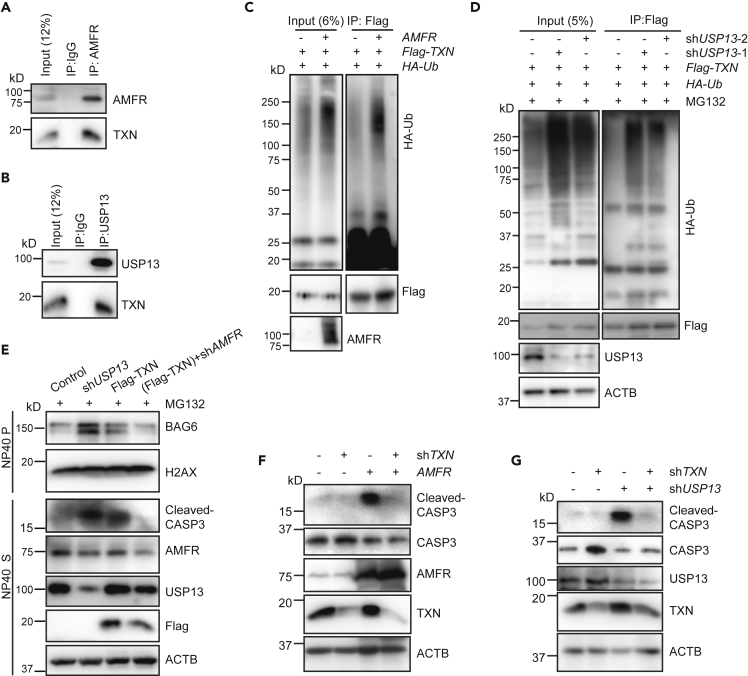
AMFR promotes TXN ubiquitination, which is antagonized by USP13. (A and B) Immunoprecipitation analysis of the interactions between endogenous TXN and AMFR (A) or USP13 (B). Immunoprecipitation using either IgG or anti-AMFR (A) or anti-USP13 (B) was performed. The co-immunoprecipitated TXN was immunoblotted with an anti-TXN antibody. (C) TXN is more ubiquitinated in *AMFR*-overexpressing cells. Cells were co-transfected with *FLAG-TXN* and *HA-ubiquitin*, along with control vector or *AMFR* construct. Ubiquitinated TXN was immunoprecipitated with anti-FLAG beads under denaturing condition, and immunoblotted with an anti-HA antibody. (D) TXN is more ubiquitinated in *USP13* knockdown cells. Cells were co-transfected with *FLAG-TXN* and *HA-ubiquitin*, along with *USP13* shRNA. Cells were treated with MG132 (10 μM) for 12 h to obtain a better ubiquitination signal. Ubiquitinated TXN was immunoprecipitated with anti-FLAG beads under denaturing condition and immunoblotted with an anti-HA antibody. (E) The cleavage of CASP3 and BAG6 is affected by USP13, AMFR, and TXN. Cells were transfected with control shRNA, *USP13* shRNA-1, *FLAG-TXN*, or (*FLAG-TXN* + *AMFR* shRNA) and treated with MG132 (10 μM) for 12 h. Samples were prepared using the same procedure as in Figure 1E and were immunoblotted with the indicated antibodies. (F and G) *TXN* depletion suppresses CASP3 cleavage induced by *AMFR* over-expression (F) or *USP13* knockdown (G). Cells were transfected with control shRNA, *TXN* shRNA, *AMFR*, or (*AMFR* + *TXN* shRNA) in (F); and control shRNA, *TXN* shRNA, *USP13* shRNA-1, or (*USP13* shRNA + *TXN* shRNA) in (G). The cleaved CASP3 was analyzed by the indicated antibody. See also Figure S3.

To validate TXN as a substrate of AMFR, we first expressed both *FLAG-TXN* and *AMFR* constructs in cells and tested the interaction of the two proteins. The result showed that FLAG-TXN could pull down AMFR in cells (Figure S3A). *In vitro* GST pulldown assay using the transmembrane-deleted recombinant AMFR (GST-AMFRΔTM) showed that it stably interacted with the recombinant His-MYC-TXN (Figure S3C). The interaction was further verified at the endogenous level using an anti-AMFR antibody to co-immunoprecipitate TXN (Figure 3A). Furthermore, our data also showed that USP13 interacted with TXN at both the overexpressing and endogenous levels (Figures S3B and 3B). Next, we examined whether TXN is a substrate of AMFR and USP13. To test this idea, *FLAG-TXN* was transfected into cells together with the *AMFR* construct and an immunoprecipitation assay using anti-FLAG beads under denaturing condition was performed. The immunoblotting result showed that TXN-conjugated ubiquitin was noticeably increased in *AMFR*-overexpressing cells (Figure 3C). Consistent with this result, TXN-conjugated ubiquitin was also accumulated in *USP13* knockdown cells (Figure 3D). These results verified that TXN is a substrate of AMFR and USP13.

We then tested the interaction of TXN and caspases. Upon induction of apoptosis by TNF-α treatment, FLAG-TXN was found to interact with CASP3, CASP7, and slight amount of CASP2 (Figure S3D). These data implied the possible activation of caspases by TXN. Interestingly, overexpression of *TXN* induced the cleavage of CASP3 and BAG6, which could be suppressed by knockdown of *AMFR*, indicating ubiquitination of TXN by AMFR might be essential for TXN denitrosylation activity (Figure 3E). On the other hand, CASP3 cleavage induced by *AMFR* overexpression or *USP13* knockdown was completely blocked when the cells were co-expressing *TXN* knockdown shRNA (Figures 3F and 3G). To further examine whether TXN reduces S-nitrosylated CASP3, we employed a biotin switch assay (Figure S3E). The biotin switch assay has been used extensively to measure protein S-nitrosylation (Forrester et al., 2009). We found a decrease of S-nitrosylated CASP3 (SNO-CASP3) in *TXN*-overexpressing cells (Figure S3F), suggesting TXN reduction of S-nitrosylated CASP3. Collectively, these results demonstrated that AMFR cooperates with USP13 to regulate the ubiquitination of TXN, which then influences S-nitrosylation of CASP3 and its activation, as well as BAG6 cleavage.

### Cleaved BAG6 Suppresses Autophagy

**Figure 4:**
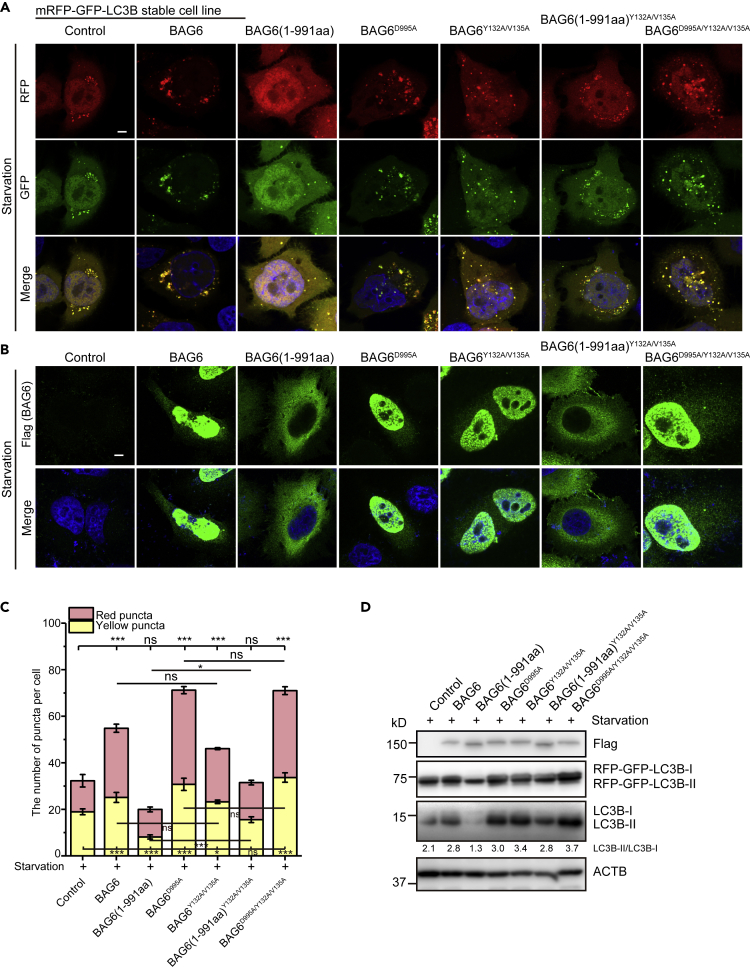
Cleaved BAG6 Remains in the Cytosol and Suppresses Autophagy (A) HeLa cells stably expressing *mRFP-GFP-LC3B* were transiently transfected with FLAG-tagged *BAG6* or its mutants. Cells were grown under starvation condition and analyzed for LC3B puncta. Nuclei are in blue. Scale bar, 5 μm. (B) Subcellular localization of BAG6 and its mutants under starvation condition. HeLa cells were transiently transfected with various FLAG-tagged Bag6 or its mutants as in (A). Cells were grown under starvation condition and stained with anti-FLAG antibody in green. Nuclei are in blue. Scale bar, 5 μm. (C) Quantitative analysis of the number of yellow (RFP^+^GFP^+^) and red (RFP^+^GFP^−^) puncta per cell as represented in (A). Data are represented as mean ± SD from three independent experiments. ∗p < 0.05; ∗∗∗p < 0.001; ns, not significant (one-way ANOVA). (D) Western blot analysis of samples from (A). mRFP-GFP-LC3B and the endogenous LC3B were examined. The LC3B-II/LC3B-I ratio was labeled to indicate the transition of LC3B under different transfection conditions. See also Figure S4.

The above results prompted us to speculate that the cleaved BAG6 is a potential regulator of autophagy and apoptosis. To see whether cleaved BAG6 could have a regulatory role in autophagy, we first transfected *BAG6* and its mutants into HeLa cells stably expressing *mRFP-GFP-LC3B*. Cells overexpressing *BAG6* greatly promoted yellow and red LC3B puncta formation under starvation condition (Figures 4A and 4C). Fluorescence imaging showed that BAG6 was exclusively localized in the nucleus (Figure 4B). In contrast, *BAG6(1-991aa)*-overexpressing cells had fewer yellow LC3B puncta compared with the control cells (Figures 4A and 4C). BAG6(1-991aa) was localized dominantly in the cytosol (Figure 4B). The caspase cleavage site mutant BAG6^D995A^, which was localized in the nucleus (Figure 4B), also induced yellow and red LC3B puncta formation similar to the full-length BAG6 (Figures 4A and 4C). The phenotype was further supported by the immunoblotting result, which showed that *BAG6* and *BAG6*^*D995A*^ promoted, whereas *BAG6(1-991aa)* inhibited, LC3B-I to LC3B-II transformation (Figure 4D).

To further address the specific importance of cleaved BAG6 in autophagic regulation, we tested *BAG6* and its mutants in *BAG6* knockout cells. Transiently expressing mRFP-GFP-LC3B was used to indicate the change of autophagy. Similarly to that observed in the wild-type background, *BAG6* or *BAG6*^*D995A*^ overexpression promoted, whereas *BAG6(1-991aa)* overexpression inhibited, yellow and red LC3B puncta formation compared with the control knockout cells (Figures S4A and S4B). These data suggest that cleaved BAG6 remains in the cytosol and prevents autophagy.

**Figure 5:**
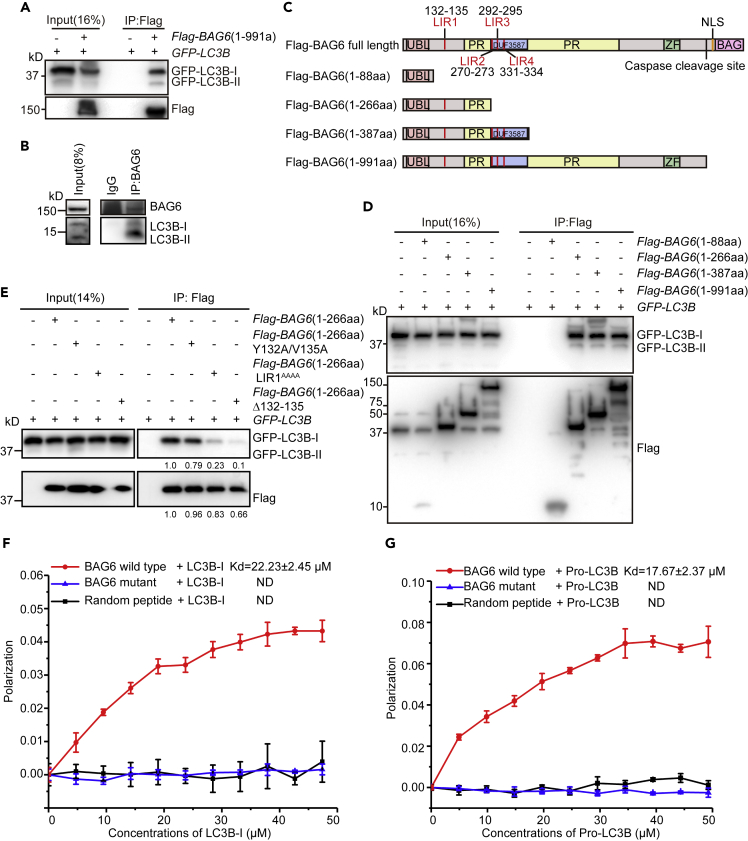
BAG6 Interacts with LC3B via a LIR Motif (A) Immunoprecipitation analysis of the interactions between FLAG-BAG6(1-991aa) and GFP-LC3B. Cells were transfected with GFP-LC3B along with the control or FLAG-BAG6(1-991aa) and FLAG pulldown was performed with anti-FLAG beads. Samples were analyzed by immunoblotting with the indicated antibodies. (B) Endogenous BAG6 interacts with LC3B in HEK293FT cells. Immunoprecipitation using either IgG or anti-BAG6 was performed. The co-immunoprecipitated LC3B was immunoblotted with an anti-LC3B antibody. (C) The domain structure of BAG6 and the LIR motifs at the N terminus of BAG6. A series of *BAG6* truncations were generated based on the domain information. (D) BAG6(1-266aa) is responsible for the interaction with GFP-LC3B. The different BAG6 truncations in (C), together with GFP-LC3B, were transfected into HEK293FT cells. FLAG pulldown was performed with anti-FLAG beads. Samples were analyzed by immunoblotting with the indicated antibodies. (E) BAG6 LIR1 mutants decreases the binding affinity to LC3B. HEK293FT cells were transfected with *GFP-LC3B*, together with FLAG-tagged wild-type *BAG6(1-266aa)*, LIR1^Y132A/V135A^, LIR1^Y132A/V133A/M134A/V135A^ (abbreviated as LIR1^AAAA^), or YVMV deletion (designated as Δ132-135). FLAG pulldown was performed with anti-FLAG beads. Samples were analyzed by immunoblotting with the indicated antibodies. The number below the blots indicates relative intensity of the proteins. (F and G) The addition of increasing concentration of LC3B-I (F) or Pro-LC3B (G) (from 0 to 50 μM) to BAG6 LIR1 peptide (1 μM) leads to a concentration-dependent increase in anisotropy value. The Kd value was calculated using nonlinear curve fitting. BAG6 LIR1 mutant and the random peptide were used as negative controls. Data are mean ± SD from three independent experiments. See also Figure S5.

We then postulated that the cleaved BAG6 may interact with the cytosolic autophagy-related proteins to regulate autophagy. We therefore employed a co-immunoprecipitation assay to screen a collection of autophagy-related proteins to see which one interacts with BAG6. We found that LC3B was co-precipitated with FLAG-BAG6(1-991aa) (Figure 5A). Thus, BAG6 may be involved in an early stage of autophagosome formation. WIPI2 is an autophagy marker for early stage, and it is recruited to nascent autophagosome by interacting with PtdIns3P at the onset of autophagosome formation (Dooley et al., 2015). Compared with the control, there was slightly more WIPI recruitment in *BAG6*- and *BAG6*^*D995A*^-overexpressing cells, but less in *BAG6(1-991aa)-*overexpressing cells (Figures S4C and S4D). These results suggest that the cleaved BAG6 suppresses autophagy at an early stage.

### BAG6 Interacts with LC3B via the LIR1 Motif

Given that BAG6 is a holdase chaperone that interacts with the hydrophobic residues of the substrate for either translocation or degradation, we suspected that the cleaved BAG6 suppresses autophagy by holding LC3 from being lipidated. We first verified that the two proteins interacted with each other at the endogenous level by co-immunoprecipitation (Figure 5B). To see which domain of BAG6 is responsible for the interaction with LC3B, we expressed a series of BAG6 truncation mutants in cells and performed co-immunoprecipitation experiments. The study identified residues 88–266aa as the minimal domain responsible for the interaction with LC3B (Figures 5C and 5D). Interestingly, residues 88–266aa contains a region that was identified previously to participate in substrate hydrophobicity recognition (Tanaka et al., 2016).

Proteins interacting with LC3 usually bear a conserved linear sequence named LIR (LC3-interacting region). LIR sequences consist of a core motif W/F/Y-X-X-L/I/V (x = acidic or hydrophobic residues) flanked by N- and C-terminal sequences (Birgisdottir et al., 2013; Johansen and Lamark, 2020). LIR-containing proteins play crucial roles in autophagy including cargo recognition, autophagosome formation and maturation, as well as many autophagy-regulated signaling pathways (Birgisdottir et al., 2013; Johansen and Lamark, 2020). By sequence analysis, we identified four putative LIR motifs in BAG6 among the region of 89–387aa (LIR1^132−135^: YVMV; LIR2^270−273^: YVEV; LIR3^292−295^: YEVL; LIR4^331−334^: FVAL) (Figure 5C). To identify the functional LIR in BAG6, we mutated each of the four LIRs and analyzed the interaction of these mutants with LC3B by co-immunoprecipitation. The result showed that FLAG-BAG6(1-266aa) LIR1^Y132A/V135A^ slightly reduced the binding affinity to LC3B. The four amino acid substitution mutant FLAG-BAG6(1-266aa) LIR1^AAAA^ and the deletion mutant FLAG-BAG6(1-266aa)Δ132-135 greatly decreased the amount of bound LC3B (Figure 5E). In contrast, mutation in LIR2/LIR3/LIR4 motifs did not affect the interaction of BAG6 and LC3B (Figure S5A).

Intriguingly, our co-immunoprecipitation studies also suggest that BAG6 might prefer to bind to LC3B-I rather than to LC3B-II (Figures 5A–5E). As shown in Figure S5B, although CQ treatment resulted in more LC3B-II, there was more LC3B-I than LC3B-II co-precipitated by BAG6. To gain more insights into the binding site between BAG6 LIR1 motif and LC3B, we synthesized an FITC-labeled 20-aa peptide encompassing the BAG6 LIR1 motif (residues 124–143) and a mutant peptide bearing two amino acid substitutions in LIR1 (LIR1^Y132A/V135A^). We also synthesized a random peptide as a negative control. We then incubated purified LC3B-I at different concentrations (up to 50 μM) with these peptides and measured fluorescence anisotropy. Indeed, addition of LC3B-I increased the anisotropy of wild-type BAG6 LIR-1 peptide but not that of BAG6 mutant peptide or the random control peptide (Figure 5F). The dissociation constant (Kd) for the binding of BAG6 LIR1 to LC3B-I was 22.23 ± 2.45 μM. These results indicate that BAG6 can directly bind to LC3B in a LIR1-dependent manner. We also introduced Pro-LC3B, the unprocessed form of LC3B, in this assay, because the protein sequence of Pro-LC3B is very similar to that of LC3B-I. Similar result was obtained with Pro-LC3B, with Kd = 17.67 ± 2.37 μM (Figure 5G). These results suggest that the cleaved BAG6 may bind to Pro-LC3B/LC3B-I and sequester them from being further lipidated.

To explore how BAG6 LIR1 affects autophagy, we transfected the *BAG6* LIR1 mutants into HeLa cells stably expressing *mRFP-GFP-LC3B*. BAG6^Y132A/V135A^ and BAG6^D995A/Y132A/V135A^ stimulated autophagy in a similar extent as their wild-type LIR1 counterparts (Figures 4A, 4C and 4D). In contrast, overexpression of *BAG6(1-991aa)*^*Y132A/V135A*^ did not suppress autophagy as that of *BAG6(1-991aa)* (Figures 4A, 4C and 4D), suggesting the involvement of LIR1 in autophagic regulation. Consistent with these results, the mutants in *BAG6* knockout cells exhibited similar phenotypes to their counterparts in the wild-type background (Figures S4A and S4B), verifying the specific role of BAG6 in autophagy. The number of WIPI2-positive dots in *BAG6(1-991aa)*^*Y132A/V135A*^-overexpressing cells was also increased compared with that in *BAG6(1-991aa)*-overexpressing cells (Figures S4C and S4D). Collectively, these results suggest that the cleaved N-terminal BAG6 regulates autophagy through its LIR1 motif.

### *BAG6* Knockdown Promotes Autophagosome Formation

**Figure 6:**
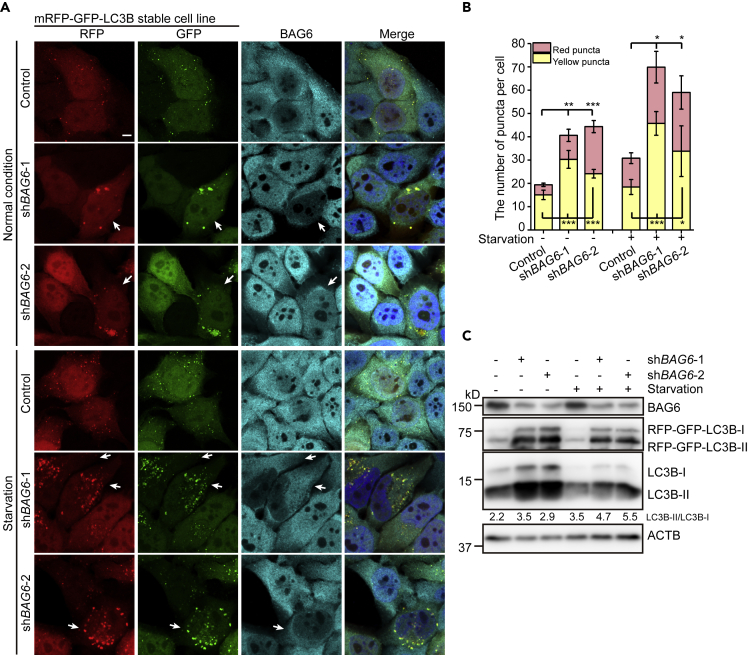
Depletion of *BAG6* Induces Autophagy (A) Depletion of *BAG6* induces aggregation of LC3B-positive puncta in cells under both normal and starvation conditions. HeLa cells stably expressing *mRFP-GFP-LC3B* were transfected with control shRNA, *BAG6* shRNA-1, or shRNA-2 and treated with normal medium or EBSS for 4 h. BAG6 is in cyan (Alexa 633) and nuclei are in blue. The arrows indicate the cells with *BAG6* knockdown. Scale bar, 5 μm. (B) Quantitative analysis of the number of yellow (RFP^+^GFP^+^) and red (RFP^+^GFP^−^) puncta per cell as represented in (A). Data are represented as mean ± SD from three independent experiments. ∗p < 0.05; ∗∗p < 0.01, ∗∗∗p < 0.001 (one-way ANOVA). (C) Western blot analysis of the samples as represented in (A). The knockdown efficiency of *BAG6* was shown. mRFP-GFP-LC3B and the endogenous LC3B were also examined. The LC3B-II/LC3B-I ratio was labeled to indicate the transition of LC3B in *BAG6* knockdown cells. See also Figure S6.

To further verify that BAG6 regulates autophagy, we performed an image-based analysis to see how depletion of *BAG6* would affect autophagy. Using the *mRFP-GFP-LC3B* stable cell line, knockdown of *BAG6* led to a noticeable increase of both yellow and red LC3B puncta number under both normal and starvation conditions (Figures 6A and 6B). Immunoblotting result further verified the transition of LC3B-I to LC3B-II under *BAG6* knockdown condition (Figure 6C). These results verified that BAG6 inhibits the autophagic flux.

Next, we examined whether autophagy attenuation caused by *AMFR* overexpression or *USP13* knockdown could be suppressed by *BAG6* knockdown. Both *AMFR* overexpression and *USP13* knockdown resulted in a decrease in the number of yellow LC3B puncta under normal and starvation conditions (Figures S6A and S6B). Interestingly, *BAG6* knockdown completely released the autophagy inhibition caused by *AMFR* overexpression or *USP13* knockdown, and the phenotype was similar to that of *BAG6* single knockdown, as indicated by the increased number of yellow and red LC3 puncta in these conditions (Figures S6A and S6B). These data suggest that BAG6 functions downstream of AMFR and USP13 in autophagic pathway.

### NMR Identifies the Direct Interaction between BAG6 LIR1 Motif and LC3B

**Figure 7:**
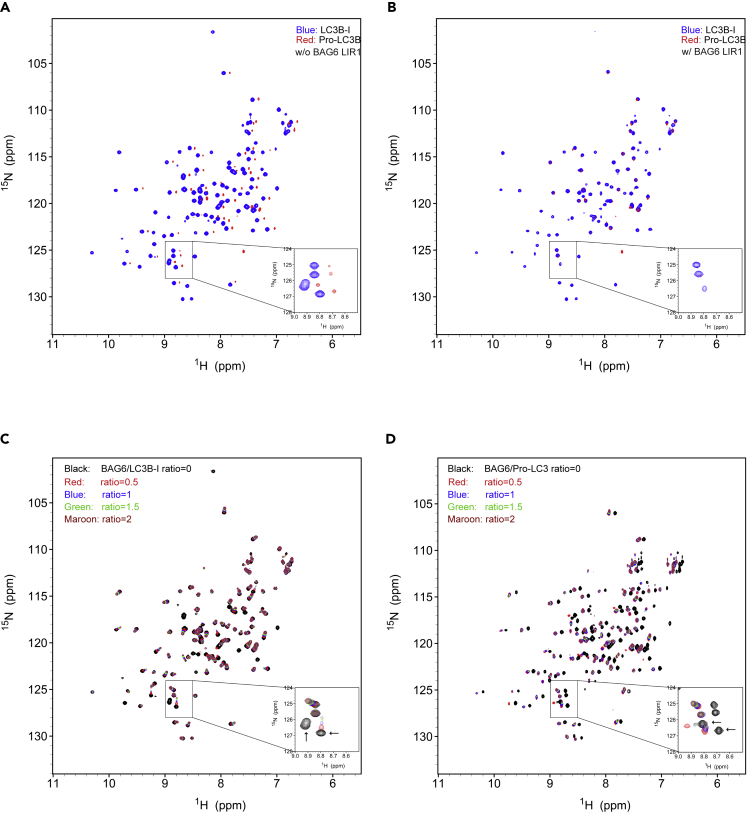
NMR Analysis of BAG6 LIR1 Motif Interacting with LC3B-I and Pro-LC3B (A) Overlay ^1^H-^15^N HSQC spectra of 0.2 mM LC3B-I (blue) and 0.2 mM Pro-LC3B without (w/o) BAG6 LIR1. The spectra were adjusted using the same contour levels to show the intensity difference between the two spectra. (B) Overlay ^1^H-^15^N HSQC spectra of 0.2 mM LC3B-I (blue) and 0.2 mM Pro-LC3B with BAG6 LIR1 at 0.5 ratio. The spectra were adjusted using the same contour levels to show the intensity difference between the two spectra. (C) Overlay ^1^H-^15^N HSQC spectra of BAG6 LIR1: LC3B-I titration at the ratio of 0 (black), 0.5 (red), 1 (green), 1.5 (blue), and 2 (maroon). Inset shows the spectrum change of four residue peaks based on BAG6 LIR1 titration. Two peaks exhibited significant broadening and finally disappeared upon BAG6 LIR1 loading (arrow). (D) Overlay ^1^H-^15^N HSQC spectra of BAG6 LIR1: Pro-LC3B titration at the ratio of 0 (black), 0.5 (red), 1 (green), 1.5 (blue), and 2 (maroon). Inset shows the spectrum change of four residue peaks based on BAG6 LIR1 titration. Two peaks exhibited significant broadening and finally disappeared upon BAG6 LIR1 loading (arrow). See also Figure S7.

To elucidate how BAG6 interacts with LC3B and the functional consequence of this interaction, we used NMR to further characterize the interaction between BAG6 LIR1 peptide and LC3B. BAG6 LIR1 and mutant peptides were synthesized and HPLC verified (Figures S7A and S7B). The ^1^H-^15^N HSQC spectra were then collected. In the absence of BAG6 LIR1, the ^1^H-^15^N HSQC spectra of LC3B-I and Pro-LC3B were different (Figure 7A). Although the concentrations of the two samples were the same, the spectra of Pro-LC3B showed weaker peak intensities. At the same time, there were differences in the peak positions. The results indicate that the two proteins adopted different structures despite significant sequence homology. The weaker spectrum profile for Pro-LC3B also suggests that Pro-LC3B may exist in several different conformational states, with only one major state showing peaks.

We then titrated different amounts of BAG6 LIR1 or BAG6 LIR1 mutant peptide into the NMR samples at the ratio of 0, 0.5, 1, 1.5, and 2 and measured the ^1^H-^15^N HSQC spectra. The spectra of Pro-LC3B changed significantly upon addition of BAG6 LIR1, but the majority of the LC3B-I peaks remained unaffected by BAG6 LIR1. Intriguingly, the HSQC spectrum of Pro-LC3B in the presence of BAG6 LIR1 at 0.5 ratio was almost identical to that of LC3B-I in the presence of BAG6 LIR1 at 0.5 ratio (Figure 7B). These results suggest that Pro-LC3B undergoes a dramatic conformational switch upon interaction with the BAG6 LIR1 and the structure of Pro-LC3B bound by BAG6 LIR1 is similar to LC3B-I. In addition, upon addition of BAG6 LIR1, some peaks in the spectra of LC3B-I (Figure 7C) and Pro-LC3B (Figure 7D) were either shifted or eliminated. For example, the insets in Figures 7C and 7D showed an expanded area where two peaks exhibited significantly broadening and finally disappeared when excess of BAG6 LIR1 was present (arrow), whereas the other two peaks only showed negligible changes upon titration of BAG6 LIR1. The disappearing residue peaks were further identified, including 4E, 12T, 19D, 33V, 51K, 52F, 53L, 81L, 85G, 96S, and 108F (Figure S7C). Most of these residues were in or near the hydrophobic pockets on the surface of LC3B, implying the location of LC3B interacting with BAG6.

Disappearance of residue peaks during the titration suggests an intermediate timescale of exchange for those residues between free and bound states. To exclude possible artifacts in the stepwise titration, solution mixtures with 0.2 mM Pro-LC3B and 0.4 mM BAG6 LIR1 were directly prepared without the stepwise titration and their HSQC spectra were also acquired. The spectra were identical to those acquired for the titration experiments (Figure S7D), indicating there were no artifacts introduced in the stepwise titration. The interaction of BAG6 LIR1 mutant with LC3B-I or Pro-LC3B was also studied using a similar NMR titration strategy. Unlike BAG6 LIR1, addition of BAG6 mutant to LC3B-I (Figure S7E) or Pro-LC3B (Figure S7F) solution caused no significant spectra change with only minor chemical shift changes for a few residues. The lack of significant disturbance in spectra indicated that this BAG6 LIR1 mutant did not interact with LC3B-I or Pro-LC3B strongly and thus confirmed that the mutations introduced into BAG6 LIR1 did inhibit its interaction with LC3B-I or Pro-LC3B. Collectively, our NMR results suggest that BAG6 LIR1 can hold Pro-LC3B or LC3B-I in a very stable state and could possibly shield LC3B to be further recruited to the autophagosome.

## Discussion

**Figure 8:**
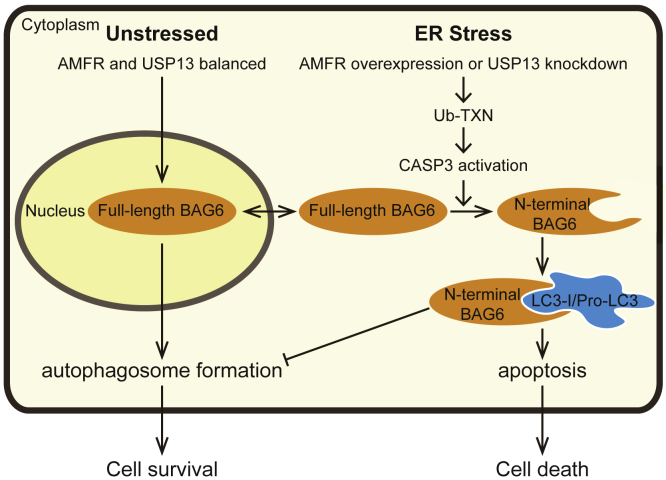
A Simple Model of BAG6 Regulation of Cellular Homeostasis between Autophagy and Apoptosis Under normal condition, BAG6 shuttles between nucleus and cytosol to keep cellular homeostasis. Under ER stress condition such as *AMFR* overexpression or *USP13* knockdown, BAG6 is cleaved by CASP3, which is activated by ubiquitinated TXN. The cleaved BAG6 is localized in the cytosol and holds LC3B to inhibit autophagy. Apoptosis is induced.

Here we showed that AMFR and USP13, previously identified E3 and DUB in the ERAD pathway, also regulate caspase activity and BAG6 cleavage to balance apoptosis and autophagy. By a powerful NEDDylation capturing system, we identified TXN as a common substrate of USP13 and AMFR and showed that TXN ubiquitination results in CASP3 activation and BAG6 cleavage. We have further discovered that BAG6 can bind to LC3B through its LIR1 motif to directly inhibit autophagosome formation. Therefore, we propose the model that BAG6 is a regulator that balances apoptosis and autophagy (Figure 8).

BAG6 has a “holdase” activity, which shields exposed hydrophobic segments of misfolded polypeptide to prevent aggregation. Given this, we questioned whether BAG6 holds autophagy-related proteins to regulate autophagy. Since we have detected BAG6 preferentially binding to LC3B-I in cells, one possibility is that BAG6 holds LC3B-I to prevent it from forming autophagosome. By sequence analysis, we found that BAG6 has a LIR1 (132–135aa) motif, which may specifically interact with LC3B. Our *in vitro* anisotropy measurement verified that BAG6 binds to LC3B-I as well as the newly synthesized Pro-LC3B with high affinity (Figures 5F and 5G). Owing to the technical difficulty to synthesize LC3B-II *in vitro*, we were not able to test whether BAG6 has lower affinity for LC3B-II. In line with this, NMR studies also showed that BAG6 LIR1 could bind to and stabilize LC3B-I structure. We found that the HSQC spectra of LC3-I with BAG6 LIR at the ratio of 0.5 remained unchanged for 10 days, whereas the HSQC spectra of LC3-I showed changes only after 2 or 3 days. Interestingly, the NMR titration experiments further showed that Pro-LC3B had two different conformational states. With the addition of BAG6 LIR1, the conformational state of Pro-LC3B changed to a state similar to LC3B-I, suggesting that BAG6 binds to Pro-LC3B to stabilize the structure similar to LC3B-I (Figures 7A and 7B). Given that endogenous full-length BAG6 shuttles between nucleus and cytosol, we propose that full-length BAG6 interacts with a limited amount of Pro-LC3B/LC3B-I, thus autophagy would not be affected. Although BAG6 gets cleaved, it remains in the cytosol and therefore holds excessive amount of Pro-LC3B/LC3B-I and prevents them to autophagosomes. In support of this hypothesis, the LIR1 mutant BAG6(1-991aa)^Y132A/V135A^ abolished its ability to inhibit autophagy (Figures 4A, 4C, and 4D). In summary, our data showed that BAG6 subcellular localization and its LIR1 are essential for its regulation in autophagy. It is also worth pointing out that it does not rule out the possibility that BAG6 can also regulate autophagy through interacting with other autophagy-related proteins.

Apoptosis and autophagy are two interconnected pathways that determine cell fate between death and survival in response to cell stress (Song et al., 2017). Common upstream signaling of these two pathways has been found (Song et al., 2017; Tsapras and Nezis, 2017). Upon ER stress, autophagy is activated preceding apoptosis to protect cells by degrading protein aggregations. Induced autophagy can block apoptosis by inhibiting the activation of caspases to reduce cellular injury, whereas autophagy can also induce apoptosis when the duration or degree of ER stress reaches the limit of cellular adaptive mechanisms. During the apoptosis stage, activated caspases can cleave autophagy-related proteins, including ATG5, BECN1 (beclin 1), and ATG4D, to inactivate autophagy (Doherty and Baehrecke, 2018; Song et al., 2017; Tsapras and Nezis, 2017). Our data showed that *USP13* knockdown inhibited autophagy, which is consistent with the previous report that USP13 promotes autophagy through deubiquitinating BECN1 to stabilize the protein (Liu et al., 2011). Interestingly, BECN1 has been reported to regulate the balance between apoptosis and autophagy (Wirawan et al., 2010; Zhu et al., 2010). BECN1 is a key component of the PtdIns3K complex, which is essential for initiation of autophagosome formation. At the onset of apoptosis, BECN1 is cleaved by caspases, and the cleaved fragments of BECN1 abolish its autophagy-inducing capacity. Instead, the C terminus of cleaved BECN1 is predominantly recruited to mitochondria to induce the release of pro-apoptotic factors (Wirawan et al., 2010). This is very similar to BAG6 in our case, for the N terminus of BAG6 holds Pro-LC3B/LC3B-I and inhibits autophagy, whereas the C terminus of BAG6 was previously proven to induce apoptosis (Wu et al., 2004).

The difficulty of identification of specific E3 substrates is the weak transient interaction between E3 and the substrates. In addition, rapid degradation of ubiquitinated substrates by proteasome also increased the difficulty. The NEDDylator system is a powerful tool to identify specific E3 substrates by modifying substrates with NEDD8 (Zhuang et al., 2013). TXN, identified by the NEDDylation system, turned out to be a common substrate of AMFR and USP13. TXN participates in the apoptotic pathway by catalyzing denitrosylation of caspases (Benhar et al., 2008; Mitchell et al., 2007; Rössig et al., 1999). S-nitrosylation is a post-translational modification, which mediates transduction of myriad cellular signals by adding nitric oxide (NO) to cysteine residues of target proteins (Sengupta and Holmgren, 2013). Previous studies demonstrated that caspases residing in mitochondria are consistently S-nitrosylated to inhibit their activity (Mannick et al., 2001). After an apoptotic stimulus, caspases are denitrosylated by TXN and rapidly activate the apoptotic pathway. Here, we found that knockdown of *USP13* increased the ubiquitination of TXN, as well as the activation of CASP3, suggesting that ubiquitination of TXN is related to the activation of CASP3. Based on these results, we propose that ubiquitination of TXN is involved in regulating TXN activity. Under normal condition, BAG6 shuttles between nucleus and cytosol to keep cellular homeostasis. Knockdown of *USP13* or overexpression of *AMFR* leads to the accumulation of ubiquitinated TXN and increased the activity of TXN. As a result, CASP3 was rapidly activated to cleave BAG6, which then holds LC3B and promotes cell apoptosis (Figure 8).

In summary, we propose a mechanism of ERAD machinery proteins, AMFR, USP13, and BAG6, in maintaining cellular homeostasis by regulating apoptosis and autophagy. The key protein BAG6 functions as a switcher to decide cell fate to either survive or die. These findings broaden our knowledge that BAG6 not only functions to help degrade client proteins but also plays a key role in balancing cellular homeostasis. Designing specific regulators of BAG6 could be a potential therapeutic strategy for its associated diseases.

### Limitations of the Study

Our study shows that BAG6 preferentially binds to LC3-I rather than LC3-II *in vivo*, and future work is needed to validate that BAG**6** inhibits LC3 lipidation *in vitro*. The new function of BAG6 LIR1 domain in autophagy needs to be further developed.

### Resource Availability

#### Lead Contact

Further information should be directed to and will be fulfilled by the Lead Contact, Yanfen Liu (liuyf@shanghaitech.edu.cn).

#### Materials Availability

Materials are available upon request from Dr. Yanfen Liu.

#### Data and Code Availability

This study did not generate/analyze datasets.

## Methods

All methods can be found in the accompanying Transparent Methods supplemental file.

## Supplemental Information

Document S1. Transparent Methods and Figures S1–S7

Table S1. Mass Spectrometry Analysis of the Substrates of NEDDylator^CUE+G2BR^ and NEDDylator^CUE^, Related to Figure 2
