# Supplementary material for: The Chaperone BAG6 Regulates Cellular Homeostasis between Autophagy and Apoptosis by Holding LC3B
Source: iScience. 2020 Oct 21;23(11):101708. doi: 10.1016/j.isci.2020.101708 (PMC7674511; doi:10.1016/j.isci.2020.101708)
Supplement: Document S1. Transparent Methods and Figures S1–S7 [file mmc1.pdf]

iScience, Volume *23*

Supplemental Information

The Chaperone BAG6 Regulates Cellular Homeostasis between Autophagy and Apoptosis by Holding LC3B

Yuanyuan Chu, Xingqi Dong, Yingjin Kang, Jingnan Liu, Tao Zhang, Cuiwei Yang, Zhangshun Wang, Wangchen Shen, Huanhuan Huo, Min Zhuang, Junxia Lu, and Yanfen Liu

Figure S1

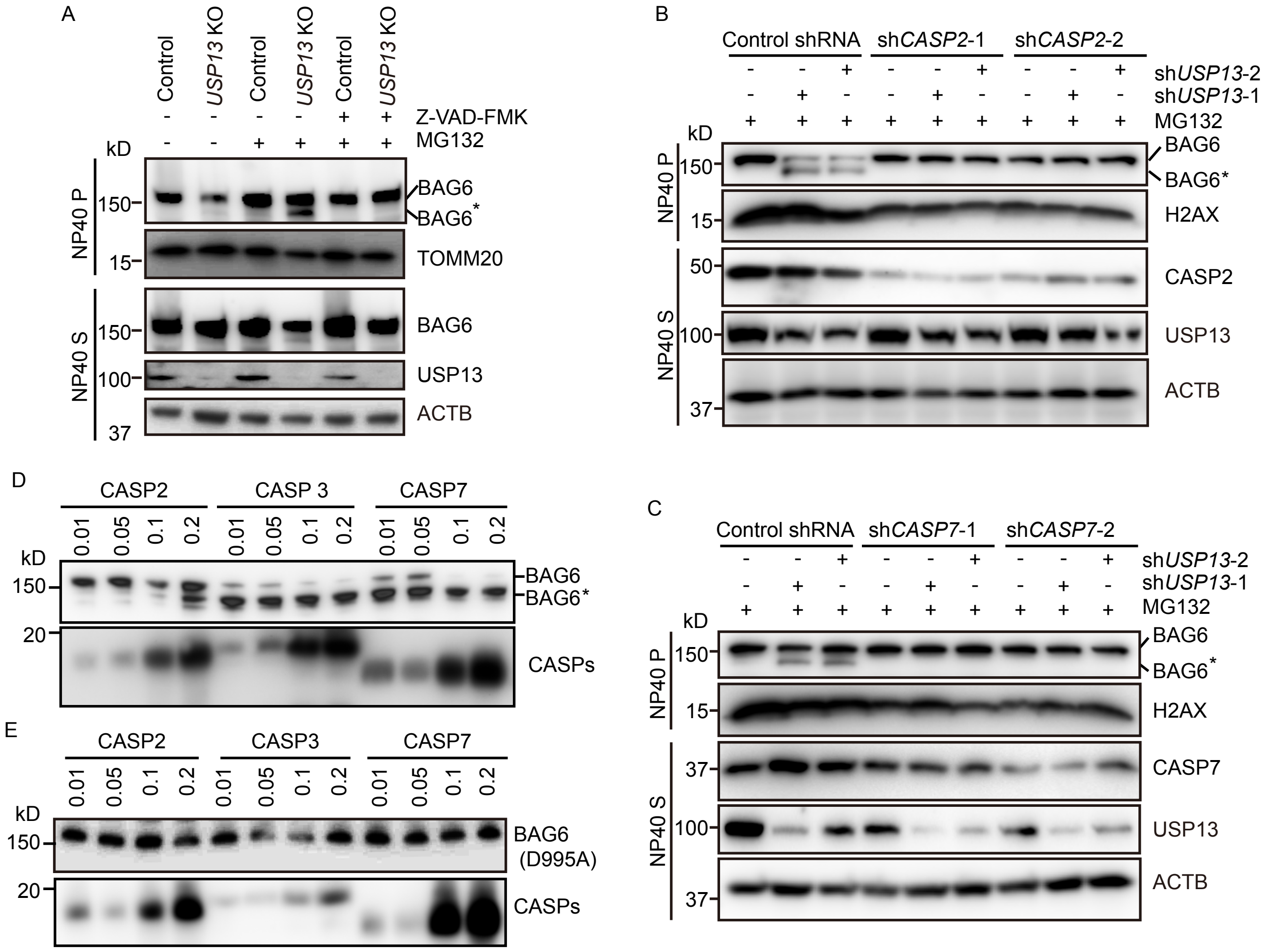
A
Control
USP13 KO
Control
USP13 KO
Control
USP13 KO
Z-VAD-FMK
MG132
kD
NP40 P
150
15
BAG6
BAG6*
TOMM20
NP40 S
150
100
37
BAG6
USP13
ACTB
B
Control shRNA
shCASP2-1
shCASP2-2
shUSP13-2
shUSP13-1
MG132
kD
NP40 P
150
15
BAG6
BAG6*
H2AX
NP40 S
50
100
37
CASP2
USP13
ACTB
D
CASP2
CASP 3
CASP7
0.01
0.05
0.1
0.2
kD
150
20
BAG6
BAG6*
CASPs
C
Control shRNA
shCASP7-1
shCASP7-2
shUSP13-2
shUSP13-1
MG132
kD
NP40 P
150
15
BAG6
BAG6*
H2AX
NP40 S
37
100
37
CASP7
USP13
ACTB
E
CASP2
CASP3
CASP7
0.01
0.05
0.1
0.2
kD
150
20
BAG6 (D995A)
CASPs

Figure S2

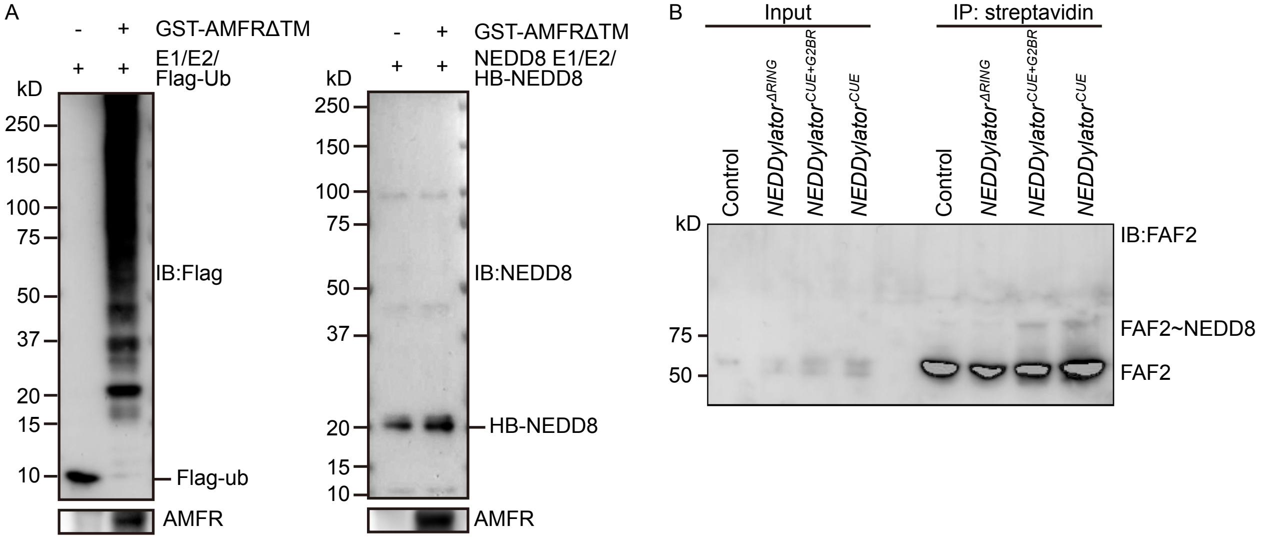
A
GST-AMFRΔTM
E1/E2/
Flag-Ub
kD
250
150
100
75
50
37
20
15
10
IB:Flag
Flag-ub
AMFR
GST-AMFRΔTM
NEDD8 E1/E2/
HB-NEDD8
IB:NEDD8
HB-NEDD8
AMFR
B
Input
IP: streptavidin
Control
NEDDylator^ΔRING
NEDDylator^CUE+G2BR
NEDDylator^CUE
IB:FAF2
FAF2~NEDD8
FAF2

Figure S3

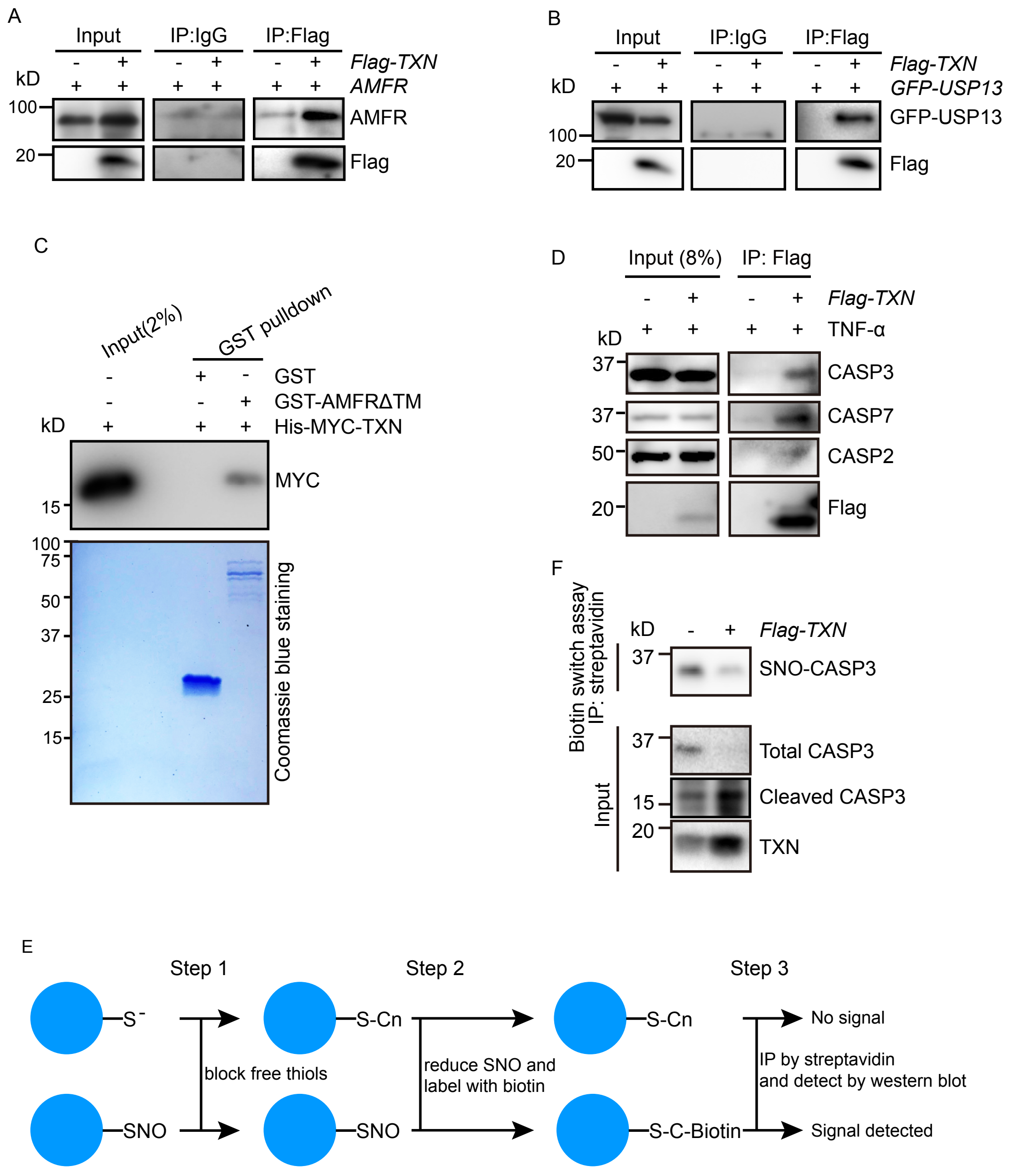

A
Input
IP:IgG
IP:Flag
Flag-TXN
AMFR
kD
100
20
AMFR
Flag
B
Input
IP:IgG
IP:Flag
Flag-TXN
GFP-USP13
kD
100
20
GFP-USP13
Flag
C
Input(2%)
GST pulldown
GST
GST-AMFRΔTM
His-MYC-TXN
kD
15
MYC
100
75
50
37
25
15
Coomassie blue staining
D
Input (8%)
IP: Flag
Flag-TXN
TNF-α
kD
37
37
50
20
CASP3
CASP7
CASP2
Flag
F
Biotin switch assay
IP: streptavidin
kD
Flag-TXN
37
SNO-CASP3
Input
37
Total CASP3
15
Cleaved CASP3
20
TXN
E
Step 1
Step 2
Step 3
S⁻
SNO
block free thiols
S-Cn
SNO
reduce SNO and
label with biotin
S-Cn
S-C-Biotin
IP by streptavidin
and detect by western blot
No signal
Signal detected

Figure S4

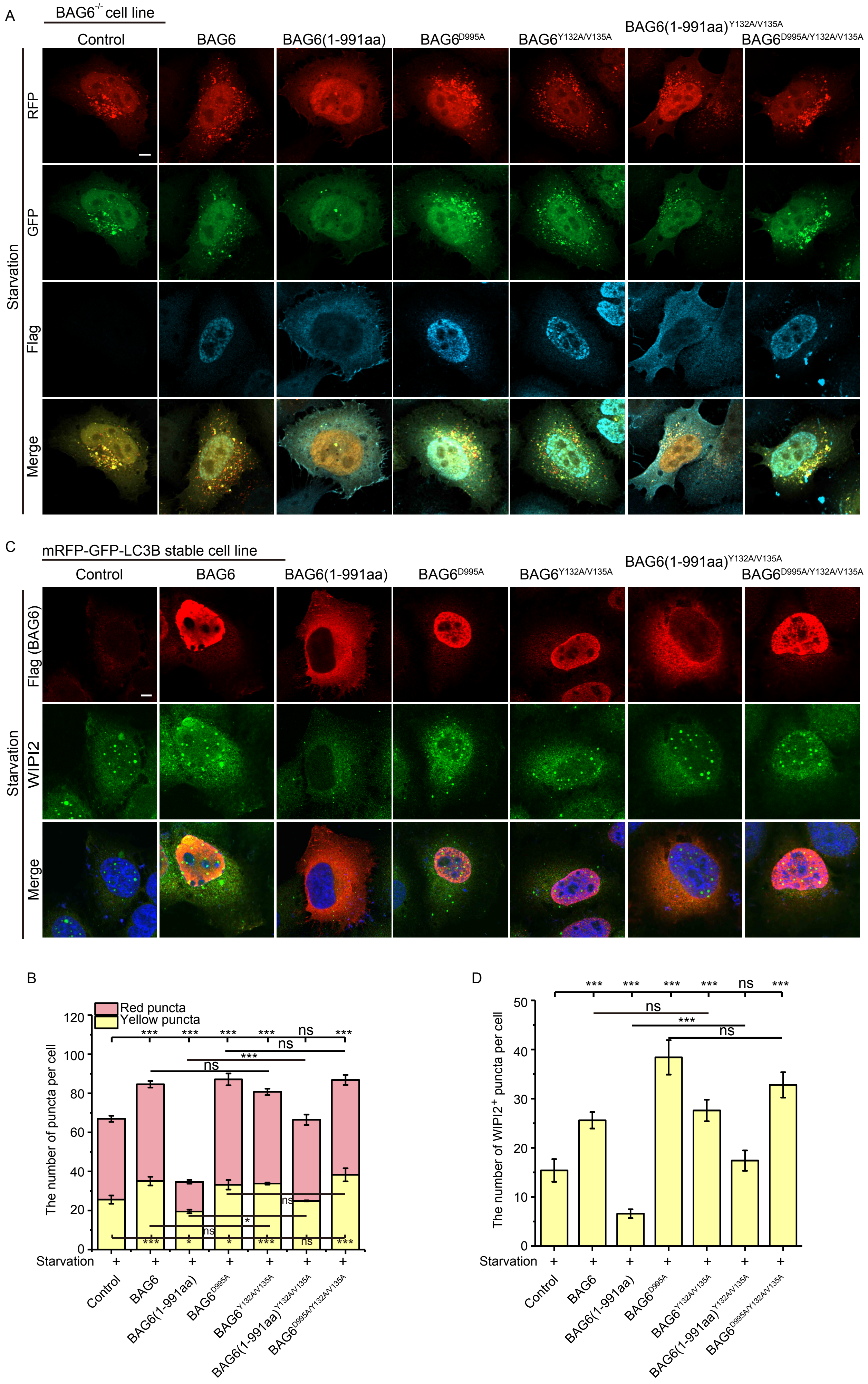

A
BAG6-/- cell line
Control
BAG6
BAG6(1-991aa)
BAG6D995A
BAG6Y132A/V135A
BAG6(1-991aa)Y132A/V135A
BAG6D995A/Y132A/V135A
Starvation
RFP
GFP
Flag
Merge
C
mRFP-GFP-LC3B stable cell line
Flag (BAG6)
WIPI2
B
Red puncta
Yellow puncta
The number of puncta per cell
D
The number of WIPI2+ puncta per cell

Figure S5

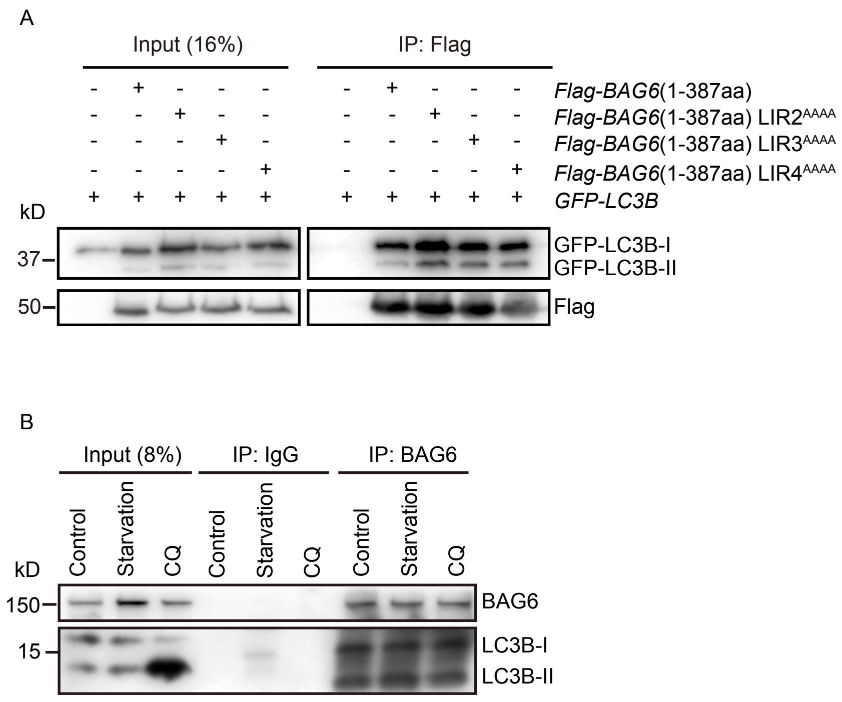
A
Input (16%)
IP: Flag
Flag-BAG6(1-387aa)
Flag-BAG6(1-387aa) LIR2AAAA
Flag-BAG6(1-387aa) LIR3AAAA
Flag-BAG6(1-387aa) LIR4AAAA
GFP-LC3B
kD
37
50
GFP-LC3B-I
GFP-LC3B-II
Flag
B
Input (8%)
IP: IgG
IP: BAG6
Control
Starvation
CQ
kD
150
15
BAG6
LC3B-I
LC3B-II

Figure S6

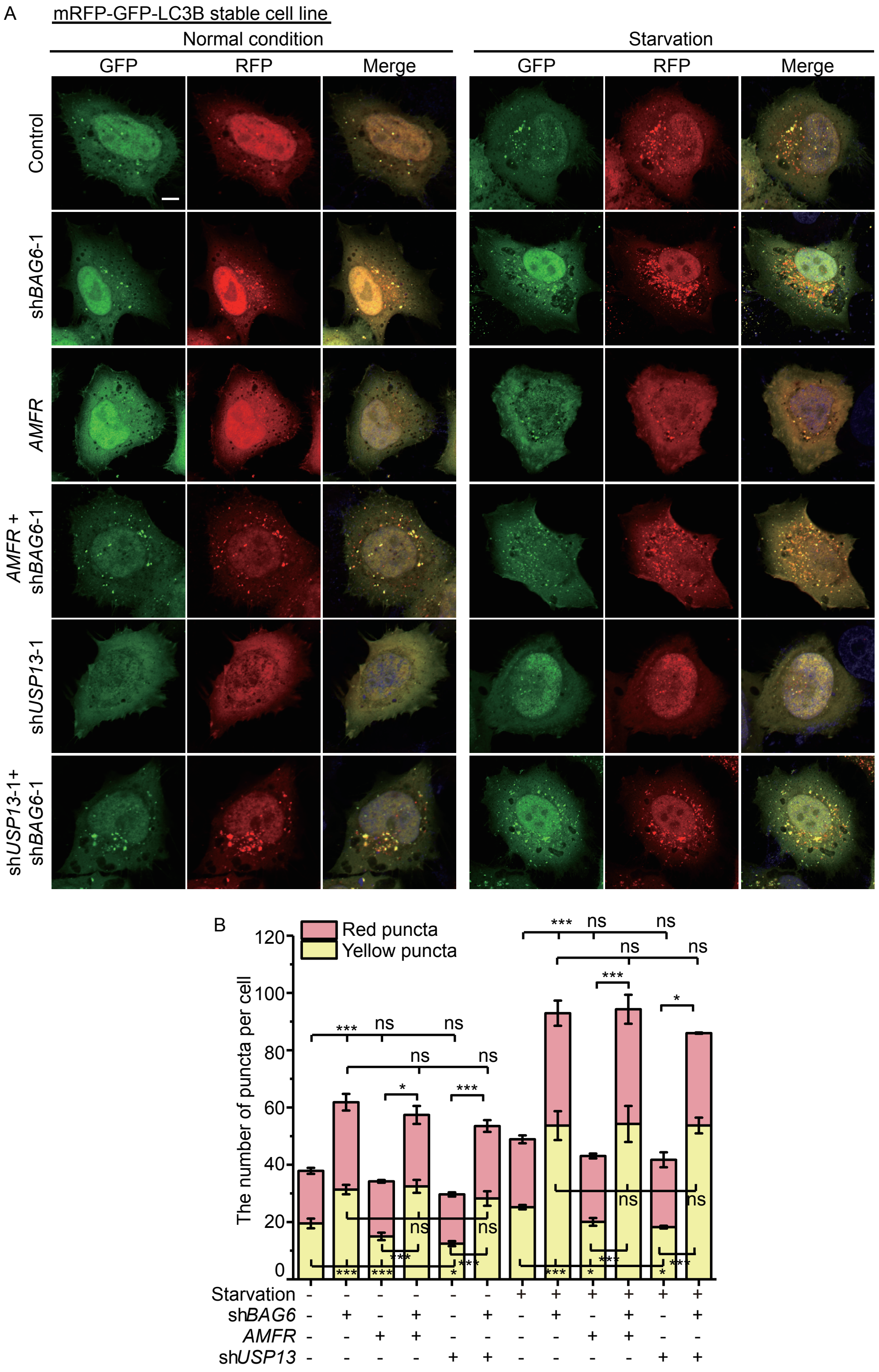

A
mRFP-GFP-LC3B stable cell line
Normal condition
Starvation
GFP
RFP
Merge
Control
shBAG6-1
AMFR
AMFR + shBAG6-1
shUSP13-1
shUSP13-1+ shBAG6-1
B
Red puncta
Yellow puncta
The number of puncta per cell
Starvation
shBAG6
AMFR
shUSP13

Figure S7

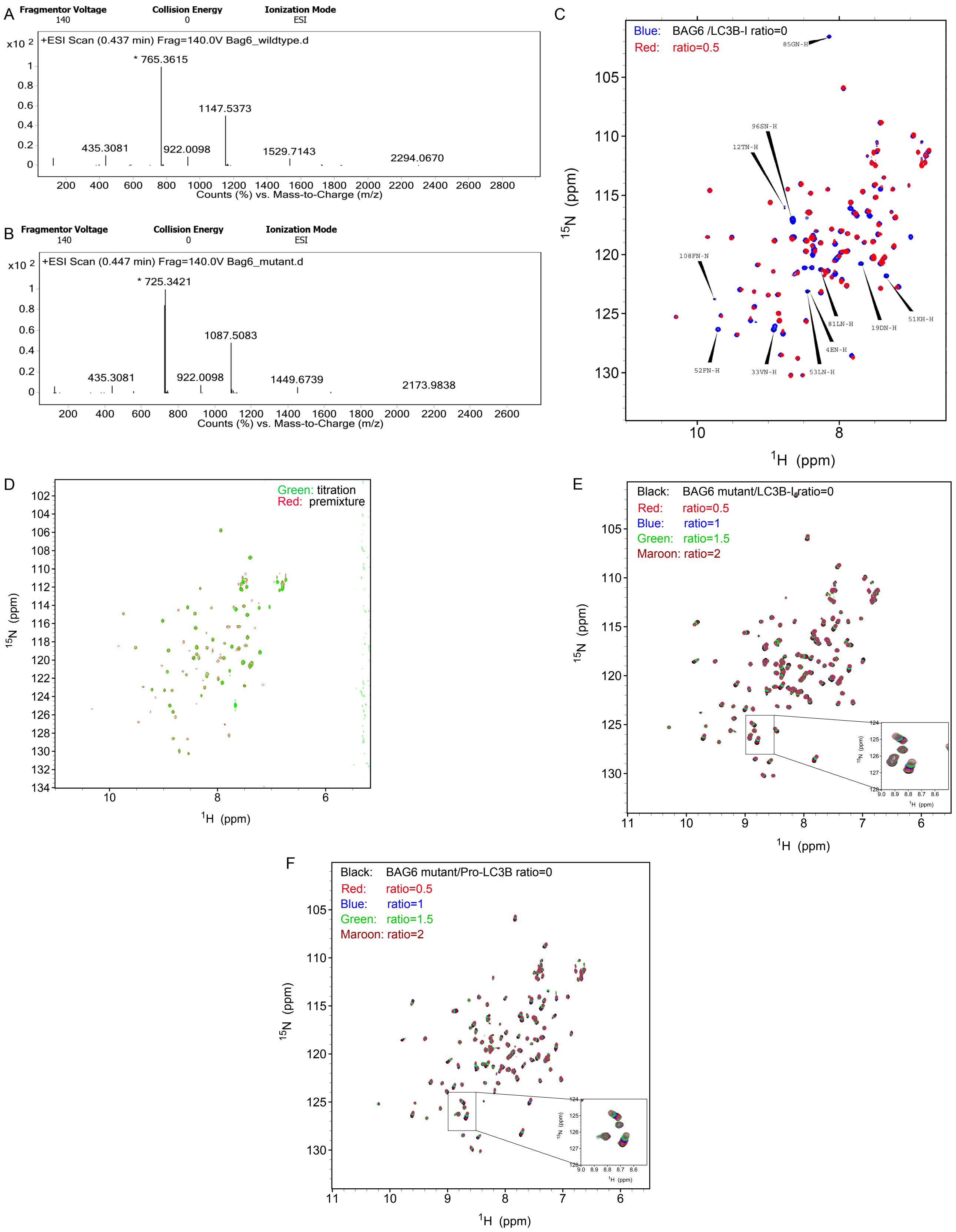

A
Fragmentor Voltage 140
Collision Energy 0
Ionization Mode ESI
x10 2
+ESI Scan (0.437 min) Frag=140.0V Bag6_wildtype.d
* 765.3615
1147.5373
435.3081
922.0098
1529.7143
2294.0670
Counts (%) vs. Mass-to-Charge (m/z)
B
Fragmentor Voltage 140
Collision Energy 0
Ionization Mode ESI
x10 2
+ESI Scan (0.447 min) Frag=140.0V Bag6_mutant.d
* 725.3421
1087.5083
435.3081
922.0098
1449.6739
2173.9838
Counts (%) vs. Mass-to-Charge (m/z)
C
Blue: BAG6 /LC3B-I ratio=0
Red: ratio=0.5
85GN-H
96SN-H
12TN-H
108FN-N
81LN-H
19DN-H
51KH-H
4EN-H
52FN-H
33VN-H
53LN-H
15N (ppm)
1H (ppm)
D
Green: titration
Red: premixture
15N (ppm)
1H (ppm)
E
Black: BAG6 mutant/LC3B-I ratio=0
Red: ratio=0.5
Blue: ratio=1
Green: ratio=1.5
Maroon: ratio=2
15N (ppm)
1H (ppm)
F
Black: BAG6 mutant/Pro-LC3B ratio=0
Red: ratio=0.5
Blue: ratio=1
Green: ratio=1.5
Maroon: ratio=2
15N (ppm)
1H (ppm)

Supplemental Figure Legends

Figure S1. Depletion of *USP13* induces BAG6 cleavage, which is blocked by caspase depletion, Related to Figure 1.

(A) The membrane fraction BAG6 was cleaved in *USP13* knockout cells which was induced by MG132 and blocked by Z-VAD-FMK. Samples were prepared using the same procedure as in Figure 1E.

(B and C) As in Figure 1F, except different caspase shRNAs were co-transfected with *USP13* shRNAs.

(D) Specific cleavage of BAG6 by recombinant purified CASP2/3/7 *in vitro*. Recombinant BAG6 was incubated with different concentrations of caspase enzymes at 37°C for 1 h.

(E) BAG6^{D995A} was not cleaved by recombinant purified CASP2/3/7 *in vitro*. As in (C), expect using the caspase cleavage site mutant BAG6^{D995A} as the substrate.

Figure S2. NEDDylation efficiency of the three NEDDylators, Related to Figure 2.

(A) Wild type AMFR exclusively mediates ubiquitination but not NEDDylation on substrates. E1-GST, His-UBE2G2 (E2), Flag-ubiquitin, and GST-AMFRΔTM were incubated for 1 h at 37°C (Left). NEDD8 E1, NEDD8 E2 UBE2M, HB-NEDD8, and GST-AMFRΔTM were incubated for 1 h at 37°C (Right). Reaction samples were stopped by adding sample buffer and analyzed by immunoblotting with Flag antibody.

(B) NEDDylator$^{CUE+G2BR}$ and NEDDylator^{CUE}, but not NEDDylator$^{\Delta RING}$, can efficiently transfer NEDD8 to a known AMFR substrate FAF2. *AMFR* knockout cells were transfected with *NEDDylator$^{CUE+G2BR}$*, *NEDDylator^{CUE}*, or *NEDDylator$^{\Delta RING}$*, along with *HB-NEDD8*. Cells were treated with biotin (5 μM) for 48 h. Samples were immunoprecipitated by streptavidin beads and analyzed by FAF2 antibody.

Figure S3. TXN interacts with USP13, AMFR and caspases, Related to Figure 3.

(A and B) Immunoprecipitation analysis of the interactions between Flag-TXN and AMFR (A), or Flag-TXN and GFP-USP13 (B). Cells were transfected with *AMFR* (A) or *GFP-USP13* (B), together with control vector or *Flag-TXN*. Immunoprecipitation using either IgG or anti-Flag was performed. The co-immunoprecipitated AMFR or USP13 was immunoblotted with an anti-AMFR (A) or anti-GFP antibody (B).

(C) GST-AMFRΔTM interacts with His-MYC-TXN. His-MYC-TXN purified from *E. coli* was incubated with GST or GST-AMFRΔTM for pulldown analysis. Samples were immunoblotted with an anti-MYC antibody.

(D) Immunoprecipitation analysis of the interactions between Flag-TXN and CASP2/3/7 under TNF-α treatment. Cells were transfected with control vector or *Flag-TXN*, and treated with TNF-α (10 ng/ml) for 24 h. Flag pulldown was performed with anti-Flag beads. Samples were analyzed by immunoblotting with the indicated antibodies.

(E) Schematic of the biotin switch labeling assay. Three major steps are involved: 1) free thiol-blocking; 2) selective reduction of S-nitrosylation (SNO) sites and labeling of resultant free thiols; 3) detection of biotin-labeled thiols.

(F) S-nitrosylated CASP3 (SNO-CASP3) is decreased in *TXN*-overexpressing cells. HEK293FT cells were transfected with control vector or *Flag-TXN*. 24 h after transfection, cells were prepared following the operation instruction of Biotin Switch Assay Kit (S-nitrosylation). After immunoprecipitation by streptavidin beads, the samples were analyzed by immunoblotting with the indicated antibodies. SNO-CASP3 level was decreased in *TXN*-overexpressing cells. The cleaved CASP3 was increased in *TXN*-overexpressing cells in the input fraction.

Figure S4. Cleaved BAG6 suppresses autophagy through its LIR1 motif, Related to Figure 4.

(A) Endogenous WIPI2 signal in HeLa cells transfected with Flag-tagged *BAG6* or its mutants. Cells were treated with EBSS for 4 h. WIPI2 is in green, Flag signal is in red, and nuclei are in blue. Scale bar, 5 μm.

(B) Quantitative analysis of the number of WIPI2 dots per cell as represented in (A). Data are represented as mean ± SD from three independent experiments. ***$p < 0.001$; ns, not significant (one-way ANOVA).

(C) *BAG6* knockout cells were transiently transfected with *mRFP-GFP-LC3B,* together with Flag-tagged *BAG6* or its mutants. Cells were treated with EBSS for 4 h, and analyzed for LC3B puncta. Flag is in cyan and nuclei are in blue. Scale bar, 5 μm.

(D) Quantitative analysis of the number of yellow (RFP^+GFP^+) and red (RFP^+GFP^-) puncta per cell as represented in (C). Data are represented as mean ± SD from three independent experiments. *$p < 0.05$; ***$p < 0.001$; ns, not significant (one-way ANOVA).

Figure S5. BAG6 interaction with LC3B is not dependent on LIR2/3/4 motifs, Related to Figure 5.

(A) BAG6 LIR2, LIR3, and LIR4 mutants do not affect the binding affinity to LC3B. Immunoprecipitation analysis of the interactions between Flag-BAG6(1-387aa) mutants and GFP-LC3B. HEK293FT cells were transfected with *GFP-LC3B,* together with *BAG6(1-387aa)*

or its LIR mutants: LIR2$^{Y270A/V271A/E272A/V273A}$ (abbreviated as LIR2^{AAAA}), LIR3$^{Y292A/E293A/V294A/L295A}$ (abbreviated as LIR3^{AAAA}), LIR4$^{F331A/V332A/L334A}$ (abbreviated as LIR4^{AAAA}). Flag pulldown was performed with anti-Flag beads. Samples were analyzed by immunoblotting with the indicated antibodies.
(B) Immunoprecipitation analysis of the interaction between BAG6 and LC3B-I/LC3B-II in HEK293FT cells. Cells were grown under normal condition, treated with EBSS for 4 h, or treated with 20 μM CQ in complete medium for 4 h. Immunoprecipitation using either IgG or anti-BAG6 was performed. The co-immunoprecipitated LC3B was immunoblotted with an anti-LC3B antibody.

Figure S6. Knockdown of *BAG6* suppresses *AMFR* overexpression- or *USP13* knockdown-induced autophagy attenuation, Related to Figure 6.

(A) Autophagy attenuation caused by *AMFR* overexpression or *USP13* knockdown is suppressed by *BAG6* knockdown. HeLa cell stably expressing *mRFP-GFP-LC3B* were transfected with control vector, *BAG6* shRNA-1, *AMFR*, (*BAG6* shRNA-1 + *AMFR*), *USP13* shRNA-1, or (*BAG6* shRNA-1 + *USP13* shRNA-1), and treated with normal medium or EBSS for 4 h. Nuclei are in blue. Scale bar, 5 μm.
(B) Quantitative analysis of the number of yellow (RFP$^+$GFP$^+$) and red (RFP$^+$GFP$^-$) puncta per cell as represented in (A). Data are represented as mean ± SD from three independent experiments. *p < 0.05; **p < 0.01; ns, not significant (one-way ANOVA).

Figure S7. NMR analysis of BAG6 LIR1 mutant interacting with LC3B-I and Pro-LC3B, Related to Figure 7.

(A and B) Identification of purified BAG6 peptide (A) or BAG6 LIR1 mutant by mass spectrometry.

(C) Identification of the disappearing residue peaks on LC3B during titration.

(D) Overlay ^{1}H-^{15}N HSQC spectrum of BAG6 LIR1: Pro-LC3B ratio=2 using titration method (Green) and a directly prepared mixture (Red).The two spectra overlay perfectly.

(E) Overlay ^{1}H-^{15}N HSQC spectra of BAG6 LIR1 mutant: LC3B-I titration at the ratio of 0 (Black), 0.5 (Red), 1 (Green), 1.5 (Blue), 2 (Maroon). Inset shows the spectrum change of four residue peaks based on BAG6 LIR1 mutant titration.

(F) Overlay ^{1}H-^{15}N HSQC spectra of BAG6 LIR1 mutant: Pro-LC3B titration at the ratio of 0 (Black), 0.5 (Red), 1 (Green), 1.5 (Blue), 2 (Maroon). Inset shows the spectrum change of four

residue peaks based on BAG6 LIR1 mutant titration.

Transparent Methods

Key Resources Table

**Oligonucleotides and peptides used in this paper**	**Source**
FITC-BAG6 LIR1 motif (VHDRNANSYVMVGTFNLPSD)	Zhengzhou Phtdpeptides Co.,Ltd
FITC-BAG6 LIR1 mutant motif (VHDRNANSAVMAGTFNLPSD)	Zhengzhou Phtdpeptides Co.,Ltd
FITC-random peptides (YGRKKRRQRRRYKEGYNVYG)	Zhengzhou Phtdpeptides Co.,Ltd
Oligonucleotides (5'-3')	
AMFR sgRNA-1: GCTAACGGCCTCGCTGCAG	This paper
AMFR sgRNA-2: GCTACACCCACGGAATGCA	This paper
USP13 sgRNA-1: AAAGACATTTTCGAAAAAC	This paper
USP13 sgRNA-2: TACATTTTTGGCCTTTGGAA	This paper
sh*BAG6* targeting sequence:<br>1. ACCGGAATGCCAACAGCTATGTCATGGTT<br>2. GCCATTCCCATACAGATCAAT	(Wang et al., 2011)
sh*USP13* targeting sequence:<br>1. CCTGAATACTTGGTAGTGCAGATAAAGAA<br>2. GCGCATGTTTAAGGCCTTTGT	(Liu et al., 2014)
sh*CASP2* targeting sequence:<br>1. GGACATCATCACCTTGGAAAT<br>2. GCACTTCACTGGAGAGAAAGA	This paper
sh*CASP3* targeting sequence:<br>1. GCGAATCAATGGACTCTGGAA<br>2. CCTGAGATGGGTTTATGTATA	This paper
sh*CASP7* targeting sequence:<br>1. GCTTCGCCTGCATCCTCTTAA<br>2. GATGGTGTCACACCAATAAAG	This paper
BAG6 Y132A/V135A mutagenic forward primer: GGAATGCCAACAGCGCTGTCATGGCTGGAACCTTCAATC	This paper
BAG6 LIR1^{AAAA} mutagenic forward primer: GGAATGCCAACAGCGCTGCCGCGGCTGGAACCTTCAATC	This paper
BAG6 Δ132-135 mutagenic forward primer: GGAATGCCAACAGCGGAACCTTCAATC	This paper
BAG6 LIR2^{AAAA} mutagenic forward primer: ATCCTTCCCCTGCGGAGGCTGCCGCGGCGCTCCAGGAGCTA	This paper

BAG6 LIR3^{AAAA} mutagenic forward primer: TTCTTGCAGCGCTACGCCGCGGCTGCGGGTGCTGCTGCCA	This paper
BAG6 LIR4^{AAAA} mutagenic forward primer: TGCTGGGCAACACCGCTGCTGCAGCGTCTGACCTGCGCT	This paper
BAG6^{D995A} mutagenic forward primer: TCCCGGGATGAACAGGCTGGAGCTTCAGCT	This paper

Cell lines

HEK293FT (PTA-5077) and HeLa cells (CRM-CCL-2) from ATCC were cultured in DMEM (Thermo Fisher Scientific, 10566024) with 10% fetal bovine serum (Gemini, 900-108) and 100 U/ml penicillin G and 100 µg/ml streptomycin (Thermo Fisher Scientific, 15140148) at 37°C under 5% CO_2. HeLa cells stably expressing *mRFP-GFP-LC3B* were cultured in complete medium containing 300 ng/µl G418 (Invivogen, ant-gn-1). CRISPR/Cas9 technology was used to generate the *USP13* and *AMFR* knockout cells. Target sequences are listed in Key Resource Table. Target sequences were inserted into *pGL3-U6-2sgRNA* plasmid. Cells were co-transfected with *pST1374-N-NLS-Flag-linker-Cas9* and sgRNA plasmids. Isolated knockout clones were verified by western blot and sequencing.

Plasmids

pCDN3.0-AMFR, *GST-AMFRΔTM*, *GFP-USP13* and *Flag-BAG6* were described previously (Liu et al., 2014). For *BAG6*, *USP13*, *CASP2*, *CASP3*, and *CASP7* shRNA knockdown, target sequences were cloned into *pSUPER.neo*. The empty vector was used as the negative control. Target sequences are listed in Key Resource Table. *pST1374-N-NLS-Flag-linker-Cas9* plasmid (Addgene, 44758) and *pGL3-U6-2sgRNA* plasmid (Addgene, 115519) were provide by Professor Xingxu Huang (ShanghaiTech University, China). Construct for *AMFR* shRNA was provided by Professor Shenyun Fang (University of Maryland, Baltimore, MD). *mRFP-GFP-LC3B* plasmid was provided by Professor Tamotsu Yoshimori (Osaka University, Japan; Addgene, 21074). *EGFP-LC3B* was constructed by inserting *EGFP-LC3B* fragment into *pcDNA3.0*. The construct for expression of *Flag-TXN* was generated by cloning the coding DNA fragment into the *pRK5-Flag* vector. The construct for expression of *His-MYC-TXN* was generated by cloning the coding DNA fragment into the *pET28a* vector. The constructs for expression of the various Flag-BAG6 truncations were generated by individually introducing a stop codon at specific positions in *Flag-BAG6* plasmid. The BAG6 LIR mutants were generated

by PCR-based mutagenesis and the mutagenic primers are listed in Key Resource Table. All mutations were confirmed by DNA sequencing. The constructs for expression of *His-Pro-LC3B* (residues 1-125) and *His-LC3B-I* (residues 1-120) were generated by cloning the coding DNA fragments into the *pET32a* vector. The constructs for expression of *His-BAG6* LIR1 motif (residues 124-143: VHDRNANSYVMVGTFNLPSD) and the mutant LIR1$^{Y132A/V135A}$ (VHDRNANSAVMAGTFNLPSD) were generated by inserting commercially synthesized oligomer into the *pET32a* vector. Mammalian expression constructs for *HB-NEDD8* and NEDD8 E2 *UBE2M*, and prokaryotic expression constructs for *HB-NEDD8*, NEDD8 E1, NEDD8 E2 *UBE2M*, *His-CASP2*, *His-CASP3* and *His-CASP7* were kindly provided by Dr. Zhuang Min (Zhuang et al., 2013). NEDDylator system plasmids were generated by fusing the different *AMFR* fragments with a NEDD8 E2 *UBE2M*.

Antibodies and reagents

Rabbit anti-USP13, rabbit anti-BAG6, rabbit anti-AMFR were described previously (Liu et al., 2014). Anti-GFP antibody was developed in rabbit using recombinant GFP protein as the immunogen. The antibody was affinity-purified on the immunizing protein immobilized on agarose. Other primary antibodies used were as follows: rabbit anti-Flag (Sigma-Aldrich, F7425); rabbit anti-CASP2 (Abcam, ab179520), rabbit anti-CASP3 (ABclonal, A2156); rabbit anti-Cleaved-CASP3 (Cell Signaling Technology, 9664); rabbit anti-CASP7 (Cell Signaling Technology, 9492); rabbit anti-TXN (Proteintech, 14999-1-AP); rabbit anti-LC3B (Sigma-Aldrich, L7543, for detecting the endogenous LC3B); mouse anti-LC3 (M186-3; for detecting the exogenous LC3B); mouse anti-ACTB (HRP-DirecT) (MBL, PM053-7); mouse anti-TOMM20 (Santa Cruz Biotechnology, sc-17764); rabbit anti-Histone H2AX (Abgent, AP20703b-400); rabbit anti-HA (Cell Signaling Technology, 3724); rabbit anti-NEDD8 (Cell Signaling Technology, 2754); mouse anti-MYC (Cell Signaling Technology, 2276); mouse anti-Ubiquitin (Santa Cruz Biotechnology, sc-8017); The secondary antibodies goat anti-mouse IgG (H+L), HRP (111-035-146) and goat anti-rabbit IgG (H+L), HRP (111-035-144) were purchased from Jackson ImmunoResearch Inc. The secondary antibodies goat anti-mouse IgG (H+L), Alexa Fluor 568 (A-11031) and goat anti-rabbit IgG (H+L), Alexa Fluor 633 (A-21071) were purchased from Thermo Fisher Scientific.

Z-VAD-FMK was purchased from Santa Cruz (sc-311561). MG132 was purchased from Medchemexpress (HY-13259). Earle's Balanced Salt Solution (EBSS) was purchased from Sigma-Aldrich (E2888).

Protein expression and purification

HB-NEDD8, CASP2, CASP3 and CASP7 were purified from BL21 cells according to previously described method (Scheer et al., 2005; Zhuang et al., 2013). Flag-BAG6 were purified using Flag affinity chromatography procedure (Sigma-Aldrich). GST-AMFRΔTM were purified from *E.coli* by GST affinity chromatography (GE Healthcare) procedure according to the reference (Russell and Wilkinson, 2005). His-Pro-LC3B (residues 1-125) and His-LC3B-I (residues 1-120) were purified using Ni-NTA beads (GE Healthcare) and then the His tag was cleaved by incubating with TEV protease at 4°C for 48 h. Free His tag and His-TEV protease were removed by Ni-NTA beads. Gel filtration size exclusive chromatography with Superdex 75 column (GE Healthcare) was employed for further purification. To purify ^{15}N-labeled proteins and ^{15}N, ^{13}C double labeled proteins, related constructs were transformed into BL21 cells. Cells were grown in LB media containing 100 μg/ml ampicillin and 34 μg/ml chloramphenicol at 37°C for 10 h. Then cells were transferred to 50 ml M9 media and cultured overnight. Next the bacterial fluid were diluted at 1:10 with M9 media and cultivated at 37°C to an OD_{600nm} around 1.0. Cells were then harvested by centrifugation at 8000 × g for 10 min at 4°C and the precipitate was transferred to^{15}N-labeled or ^{15}N, ^{13}C double labeled M9 media and cultivated at 37°C to an OD_{600nm} around 1.0. Expression of isotope labeled proteins were induced with 0.5 mM IPTG at 16°C overnight. Proteins were purified through Ni-NTA beads using the same procedure as describe above. For purification of His-BAG6 LIR1 motif (residues 124-143) and its mutant His-BAG6 LIR1$^{Y132A/V135A}$, the polypeptides were further purified with HPLC after being cleaved by TEV protease.

GST pulldown assay, immunoprecipitation, and immunoblotting

GST-tagged proteins were incubated with GST beads for 1 h in PBS buffer, and the unbound proteins were removed by centrifugation at 1000 × g for 3 min. Next, GST-tagged protein bound beads were incubated with target proteins in NP40 lysis buffer (150 mM sodium chloride, 0.5% NP40 and 50 mM Tris–HCl pH 7.4) for 1 h. Then GST beads were washed three times with NP40 wash buffer (150 mM sodium chloride, 0.1% NP40 and 50 mM Tris–HCl pH 7.4). The samples were eluted by 1 × sample buffer and detected by western blot. For immunoprecipitation experiments, related plasmids were transfected into HEK293FT cells and were extracted by NP40 lysis buffer containing protease inhibitor cocktail. The soluble supernatant fractions were harvested by centrifugation at 17000 × g for 10 min and then used for immunoprecipitation with indicated antibodies. After incubating for 1 h, beads were washed three times with NP40 wash buffer, and then detected by western blot. For immunoprecipitation under denaturing condition, harvested cells were lysed in a buffer with 1% SDS and 5 mM DTT.

The samples were heated at 65°C for 10 min and diluted into 0.1% SDS and 0.5 mM DTT with NP40 lysis buffer. The soluble supernatant fractions were harvested and subjected to immunoprecipitation experiments as described above. Immunoblotting was then performed using polyvinylidene fluoride membrane (Bio-Rad, 1620177) and the indicated antibodies. ECL western blotting detection reagents (PerkinElemer, NEL105001EA) was used to detect the protein signal and the chemiluminescence bands were imaged under Amersham Imager 600 (GE Healthcare Life Sciences, USA). The bands were adjusted within the linear range, and quantified by ImageJ software (NIH).

Immunofluorescence microscopy

HeLa cells stably expressing *mRFP-GFP-LC3B* were seeded on small glass slides and transiently transfected with the indicated constructs. The small glass slides were collected 24 h after transfection and fixed with PBS containing 4% paraformaldehyde and 4% sucrose. After being washed for three times with PBS, the nuclei were labeled with DAPI (Sigma-Aldrich). Finally the samples were mounted with ClearMount™ Mounting solution (Invitrogen, USA). For the immunofluorescence stain studies, fixed cells were washed for four times with PBS, and then incubated with the indicated primary antibodies (1:500) for 1 h, and fluorescent dye-conjugated secondary antibodies (1:600) for 30 min. Images were acquired on Zeiss LSM800 or LSM880 microscope (Zeiss, Germany) with a 63 × 1.4 NA oil objective. Same acquisition parameters were used for a specific set of experiments.

Cell viability assay

Cell viability was measured by a cell proliferation assay kit (CellTiter 96® AQueous One Solution) (Promega, G3582), following the manufacturer's instructions. HeLa cells transfected with control shRNA or *USP13* shRNA were seeded in a 96-well plate. Cell death was measured by utilizing CellTiter 96® AQueous One Solution Reagent (contains a novel tetrazolium compound [3-(4,5-dimethylthiazol-2-yl)-5-(3-carboxymethoxyphenyl)-2-(4-sulfophenyl)-2H-tetrazolium, inner salt; MTS] and an electron coupling reagent (phenazine ethosulfate; PES)) added to cells for 3 h at 37°C. Absorbance was recorded at 490 nm using a 96-well plate reader (MD SpectraMax i3, USA). Three independent experiments were performed.

NEDDylator system

The NEDDylator system were constructed according to reference (Zhuang et al., 2013). Briefly, NEDDylator were generated by fusion expressing substrate binding domain of AMFR with NEDD8 E2 UBE2M. The *NEDDylator* and *HB-NEDD8* were co-transfected into *AMFR* knockout cells. After being cultured in DMEM containing 5 μM biotin for 48 h, cells were harvested and subject to immunoprecipitation with Ni-NTA column. The bound proteins were then eluted by

low pH buffer and subjected to another round of immunoprecipitation by Pierce™ Streptavidin Magnetic Beads (Thermo Fisher Scientific, 88816). Proteins were then analyzed by mass spectrometry.

Biotin switch assay

HEK293FT cells were transfected with the indicated constructs. 24 h after transfection, cells were prepared following the operation instruction of Biotin Switch Assay Kit (S-nitrosylation) (Abcam, ab236207) (also see Figure S3E). First, cells were washed three times with S-nitrosylation wash buffer. Then cells were lysed and blocked with Buffer A containing blocking reagent for 30 min at 4°C (Figure S3E, step 1). The clarified lysates were harvested by centrifugation at 17000 × g for 10 min and the proteins in supernatants were precipitated by ice-cold acetone for 1 h at -20°C. Next the protein pellets were incubated with Buffer B containing reducing and biotin labeling reagents for 1 h (Figure S3E, step 2). Then the proteins were precipitated again by ice-cold acetone for 1 h at -20°C, followed by resuspension with cold S-nitrosylation wash buffer. To detect the S-nitrosylated proteins, samples were immunoprecipitated by streptavidin beads and then immunoblotted with anti-CASP3 antibody to examine the level of S-nitrosylated CASP3 (SNO-CASP3) (Figure S3E, step 3).

Anisotropy measurement

FITC-labeled BAG6 LIR1 peptide, BAG6 LIR1 mutant peptide and random peptide were commercially synthesized. These peptides were pre-dissolved in HEPES buffer (150 mM sodium chloride and 10 mM HEPES, pH 7.4) containing 10% dimethyl sulfoxide (DMSO) as a concentrated stock solution. For anisotropy measurement, BAG6 peptides or random peptide were diluted to 1 μM with HEPES buffer and mixed with LC3B-I or Pro-LC3B range from 0 μM to 50 μM. Anisotropy of the mixture was detected using a HORIBA FluoroMax-4 (Japan) with the fluorescence polarization module. Excitation wavelength was 494 nm and emission was detected at 518 nm. Data analysis was conducted using OriginPro (Origin lab cooperation, USA) as well as Microsoft Excel Data Analysis package. The Kd value for the binding was calculated using nonlinear curve fitting in OriginPro.

NMR titration

NMR experiments were performed at 25°C on Agilent 800 MHz spectrometer and 600 MHz spectrometers equipped with a Cryoprobe. 0.2 mM ^{15}N-labeled Pro-LC3B and 0.2 mM ^{13}C,^{15}N-labeled LC3B-I protein were prepared in 50 mM Tris-HCl pH 7.5, 100 mM sodium chloride buffer with 10% D_2O. 4 mM wild type BAG6 LIR1 peptide or its mutant was pre-dissolved in 50 μl dimethyl sulfoxide (DMSO) as a concentrated stock solution. For titration experiments, the concentration ratio of BAG6 LIR1 peptide to LC3B-I or Pro-LC3B was controlled in the range from 0 to 2 by adding extra volume of BAG6 LIR1 peptide solution into the mixture. 2D ^{1}H-^{15}N HSQC experiments were carried out with 32 scans, acquiring 1562 (800 MHz spectrometer) or 1024 (600 MHz spectrometer) points in the direct dimension (^{1}H dimension), and 128 points in the indirect dimension (^{15}N dimension). DDS is used to determine 0 ppm of H dimension. All NMR spectra were processed using NMRPipe (Delaglio et al., 1995) and analyzed with Sparky (Goddard and Kneller).

Quantification and statistical analysis

Data are representative of at least three independent experiments. For quantitative analyses, values were expressed as mean ± SD, and error bars represented the standard deviations from counting of 80 cells in each group from three independent experiments. Statistical analyses were performed by using OriginPro and Microsoft Excel Data Analysis package. The significance among multiple groups were obtained using one-way ANOVA followed by Tukey's multiple comparisons test. ns, not significant; *, $p < 0.05$; **, $p < 0.01$; ***, $p < 0.001$.

Supplemental References

Delaglio, F., Grzesiek, S., Vuister, G.W., Zhu, G., Pfeifer, J., and Bax, A. (1995). NMRPipe: a multidimensional spectral processing system based on UNIX pipes. J. Biomol. NMR *6*, 277-293.

Goddard, T.D., and Kneller, D.G. SPARKY 3, University of California, San Francisco.

Liu, Y., Soetandyo, N., Lee, J.G., Liu, L., Xu, Y., Clemons, W.M., Jr., and Ye, Y. (2014). USP13 antagonizes gp78 to maintain functionality of a chaperone in ER-associated degradation. Elife *3*, e01369.

Russell, N.S., and Wilkinson, K.D. (2005). Deubiquitinating enzyme purification, assay inhibitors, and characterization. Methods Mol. Biol. *301*, 207-219.

Scheer, J.M., Wells, J.A., and Romanowski, M.J. (2005). Malonate-assisted purification of human caspases. Protein Expr. Purif. *41*, 148-153.

Wang, Q., Liu, Y., Soetandyo, N., Baek, K., Hegde, R., and Ye, Y. (2011). A ubiquitin ligase-associated chaperone holdase maintains polypeptides in soluble states for proteasome degradation. Mol. Cell *42*, 758-770.

Zhuang, M., Guan, S., Wang, H., Burlingame, A.L., and Wells, J.A. (2013). Substrates of IAP ubiquitin ligases identified with a designed orthogonal E3 ligase, the NEDDylator. Mol. Cell *49*, 273-282.
